# Assessment of pathological response to therapy using lipid mass spectrometry imaging

**DOI:** 10.1038/srep36814

**Published:** 2016-11-14

**Authors:** Nathan Heath Patterson, Balqis Alabdulkarim, Anthoula Lazaris, Aurélien Thomas, Mieczyslaw M. Marcinkiewicz, Zu-hua Gao, Peter B. Vermeulen, Pierre Chaurand, Peter Metrakos

**Affiliations:** 1 Department of Chemistry, University of Montreal, Montreal, Quebec, Canada; 2 Department of Surgery, McGill University Health Center, Quebec, Canada; 3 Cancer Research Program, McGill University Health Center-Research Institute, Quebec, Canada; 4 Unit of Toxicology, University Centre of Legal Medicine, Geneva-Lausanne, Switzerland; 5 Faculty of Biology and Medicine, Lausanne University Hospital, University of Lausanne, Lausanne, Switzerland; 6 Cytochem Inc., 6465 Durocher Avenue, Montreal, Quebec, Canada; 7 Department of Pathology, McGill University Health Center, Quebec, Canada; 8 Translational Cancer Research Unit, GZA Hospitals St.-Augustinus, 2610, Wilrijk, Belgium

## Abstract

In many cancers, the establishment of a patient’s future treatment regime often relies on histopathological assessment of tumor tissue specimens in order to determine the extent of the ‘pathological response’ to a given therapy. However, histopathological assessment of pathological response remains subjective. Here we use MALDI mass spectrometry imaging to generate lipid signatures from colorectal cancer liver metastasis specimens resected from patients preoperatively treated with chemotherapy. Using these signatures we obtained a unique pathological response score that correlates with prognosis. In addition, we identify single lipid moieties that are overexpressed in different histopathological features of the tumor, which have potential as new biomarkers for assessing response to therapy. These data show that computational methods, focusing on the lipidome, can be used to determine prognostic markers for response to chemotherapy and may potentially improve risk assessment and patient care.

Many solid tumors (colon cancer, breast cancer, gastric cancer, oesophageal cancer etc.) receive neoadjuvant chemotherapy prior to resection and the prognosis of the patient as well as the adjuvant therapeutic strategy is determined by the pathologic response to the neoadjuvant regime. Thus, the development of objective and quantitative strategies to evaluate pathologic response is important in many solid tumors.

Despite advances in management of colorectal cancer liver metastasis (CRCLM) patients, the majority are incurable^1^,^2^,^3^. Surgical resection is the only chance for cure but only feasible in 15–20% of patients^4^,^5^. Various clinical parameters such as the number and size of lesions, disease free interval, and serum Carcinoembryonic antigen levels, among others have been used to prognosticate these patients. However, with modern day therapies including preoperative chemotherapy and loco-regional modalities, these factors no longer correlate effectively with post-operative disease free and overall survival rates^6^,^7^,^8^,^9^. Similarly, clinical scoring systems that utilize these parameters are no longer appropriate, in that even patients with a poor clinical risk score that have a good response to neoadjuvant chemotherapy will have a better outcome than the clinical risk score would have predicted^10^,^11^. There are only a few molecular biomarkers that can predict prognosis and guide treatment^12^,^13^. These include KRAS that is used to select patients for treatment with anti-EGFR agents and microsatellite instability that is a prognostic factor and a marker for response to 5-FU^14^,^15^.

Pathological evaluation can predict patient outcome by assessing response to preoperative chemotherapy but can also evaluate surrounding unaffected liver parenchyma for chemotherapy associated toxicity that could lead to morbidity, further driving the choice of future chemotherapy and patient management^16^,^17^. Pathological grading systems have been developed to address this with variable correlation with survival^18^,^19^,^20^,^21^,^22^. Early post chemotherapy grading systems looked at percent viable tumor cells and more recently the Tumor Regression Grade (TRG)^21^ demonstrates a correlation with patient outcomes. This grading takes into consideration the degree of fibrosis and necrosis in relation to tumor viability, in which fibrosis is considered a positive indicator and necrosis a negative indicator of response^20^. However with the widespread use of Bevacizumab, an angiogenesis inhibitor precipitating tumor necrosis as a form of response, the Modified Tumor Regression Grade (mTRG) emerged^23^. mTRG differentiates between two types of necrosis seen in resection specimens; usual necrosis (UN), typical of tumor progression, and infarct-like necrosis (ILN), a response to treatment, whose presence has been associated with favourable prognosis^23^. However, the pathological evaluation process remains a subjective semi-quantitative one regardless of which grading system is applied and is subsequently vulnerable to inter-pathologist variability necessitating a complementary technical approach^24^,^25^. In addition, the final pathological report can take between 7–14 days, depending on the institution.

We propose to compliment pathologic assessment of CRCLMs with matrix-assisted laser desorption/ionization (MALDI) mass spectrometry imaging (MSI). MSI images biomolecules including proteins and lipids directly on thin tissue sections of clinical origin maintaining spatial orientation^26^,^27^,^28^,^29^. MSI acquires mass spectra at defined 2-D positions across a surface and reconstructs these data into ion distribution maps reflecting molecular distributions^30^,^31^. Phospholipids are of clinical interest due to their biological roles in both physiological states and cancer processes^32^. The potential clinical utility of phospholipid MSI has been demonstrated in a number of applications^32^,^33^,^34^,^35^, however, tissue studies with large cohorts still require robust and validated approaches for data interpretation due to the highly multivariate nature of MSI datasets and the inherently complex nature of biological tissue^36^. Currently, two approaches exist for analyzing clinical MSI data: Histology-driven MSI approaches where one determines MSI regions of interest based on histopathology annotations of stained serial sections, and histology-independent approaches where regions of interests are determined from multivariate molecular patterns inherent to the MSI data^37^. Upon isolating and validating signal patterns found in tissue, MSI offers the capability to automatically classify the topography of new samples^38^. When a sufficient spectral library has been generated and correlated with pathology and/or clinical outcomes, MSI data can then be further mined for potential disease biomarkers. Thus MSI can complement a subjective pathology assessment where histopathological areas are defined using a computational, less prone to human bias, assessment of pathological areas within tissue. Equally important is that this methodology can generate a numerical output within a fraction of the time that it takes to deliver a conventional, semi-quantitative pathology report in most clinical institutions.

Here we describe the use of MSI to grade response in clinical CRCLM tissues. Importantly, the MSI generated grading reflected disease progression and overall survival. In addition, we demonstrate that this methodology can identify unique lipid distributions and moieties that could potentially be used as prognostic markers, therapeutic targets, and help in further understanding the biology of CRCLM.

## Results

### Workflow employed in the study

**Figure 1:**
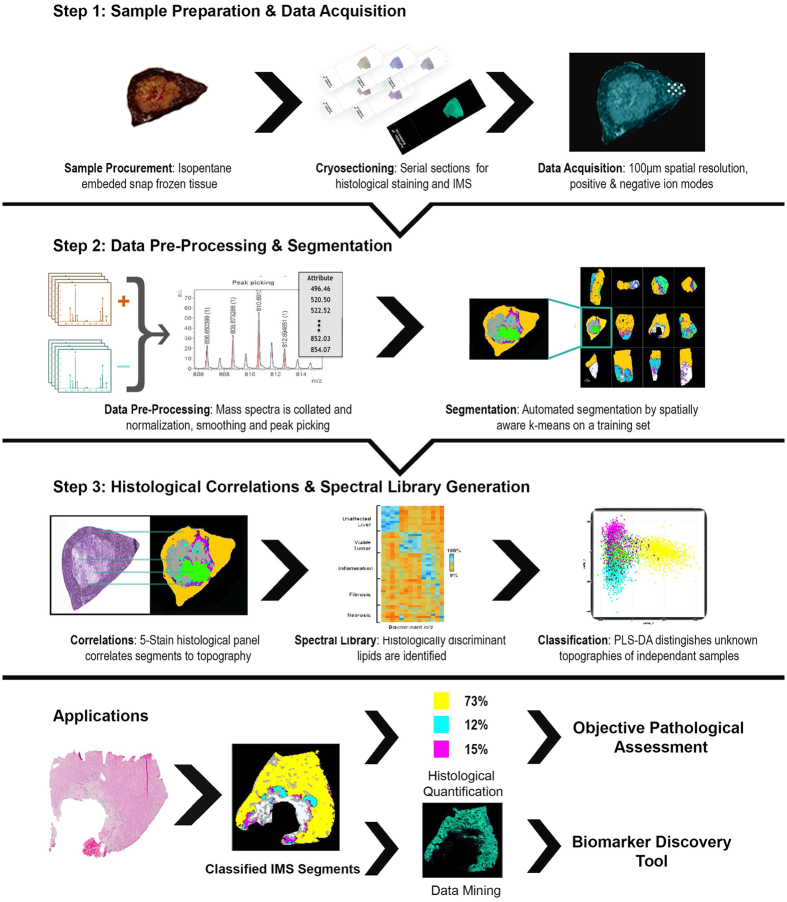
Summary of workflow. a.i. = arbitrary intensity.

Our workflow is briefly outlined in Fig. 1: it begins with MSI data acquisition from CRCLM specimens in both positive and negative ionization mode (step 1). The raw spectral data is pre-processed by total-ion-current (TIC) normalization and then peak picked followed by spatially-aware k-means segmentation of the peak data. (step 2). The generated segments are correlated visually with the major histological compartments within the sample using multiple stained serial sections of the tissue. The correlated segments are trained to partial least squares–discriminant analysis (PLS-DA) algorithm. This completes the histology driven part of the workflow, ending with a spectral library and PLS-DA classifier (step 3). The lipid signatures are then validated by classifying an independent cohort of specimens. The resulting classification can be used to compute an mTRG grading for direct clinical application that is less subjective than classic pathology. Finally, while multivariate analysis of the data allows classification of new samples, univariate examination of the data identifies histologically discriminating species with potential clinical or fundamental impact.

After development of a training set, sample throughput is limited to the time of sample preparation, data acquisition, pre-processing and classification. Sample preparation for one sample including cutting and matrix sublimation should take anywhere from 30–90 minutes. Data acquisition on the instrument used in this study was at a speed of 2 pixels/s, generally around 1 to 2 hours for an average sized sample. Next generation MALDI systems are routinely capable of 25+ pixels/s with some up to 50 pixels/s. At this acquisition speed a sample of 1 cm^2^ at 100 μm of spatial resolution would take only a matter of minutes. Finally, data processing including normalization and peak picking will take approximately an hour.

### Correlation of Histopathology to Mass Spectrometry Imaging

Our first goal was to determine whether signatures derived from MSI data are capable of robustly distinguishing different histopathological features of CRCLM tumor specimens. To achieve this, a training set of 12 CRCLM specimens from 12 patients were analyzed by MSI using sublimated DAN matrix with no sample preparation beyond cutting and desiccation of the tissue sections. All MSI results are only relatively quantitative in the mass spectrometric sense; no internal standards have been used. After pre-processing, the acquired MSI spectra were segmented using spatially aware k-means. This algorithm incorporates spatial data into the clustering analysis by considering similarity between neighbouring pixels during computation. The algorithm is implemented in the Cardinal R package, and we used a pixel radius of 1 for all segmentation. The generated segments were then correlated to tumor morphology by visual comparison with H&E-stained sections of the same specimens. This methodology required expert pathology in order to determine the best segment correlation for each histological region. Using 100 micron MSI resolution, gross tissue regions were assessed rather than the intricacies apparent through optical microscopy of stained sections. Liver lesions ranged in size from 0.3 to 9.2 cm with a mean size of 4.7 cm. The mean follow up for this cohort of patients was 30.2 months. Overall survival (OS) and disease free survival (DFS) by the end of the study period in this group of patients was 81.8% and 48.6% respectively. Relevant clinical data is presented in supplementary Table S1 and Figure S1. The clinical data highlights the diversity of the cohort.

**Figure 2:**
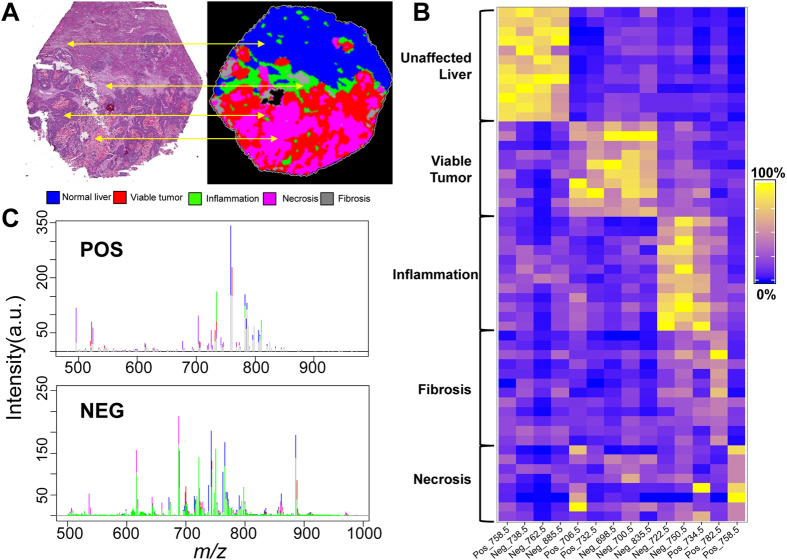
Correlation of MSI to histological staining and mining MSI data. (**A**) Visualization of the workflow employed comparing MSI segmentations to staining of serial sections. Color scheme of various detected regions is given below. (**B**) Tile plot of signal intensity of averages of 14 most discriminant markers from MSI listed by correlated histology. (**C**) Average of training set peak intensity from all pixels within the correlated histology from positive and negative ionization modes, top and bottom, respectively. Colors are as presented in section A. (a.u.) = arbitrary units.

Figure 2 illustrates MSI segmentation of a tissue section from the training set, visually correlated to an H&E stain of the same section presented here at low magnification (2A). Segmentation of the data into 7 molecular signatures (k = 7) was able to identify 6 histopathological features of the tumor and surrounding areas within the training set, as one cluster defines MALDI matrix noise. We have color coded the 6 signatures (a signature is defined as a combination of lipids and intensity unique to a histopathology) as: adjacent unaffected liver (blue), tumor (red), necrosis (purple), fibrosis (grey), inflammation (green) and mucin/MALDI matrix noise (white). Segmentation algorithms provide an unsupervised means of data reduction and when correlated to histology, prevent analysis of irrelevant MSI pixels that may occur with manual selection of ROIs in spatially heterogeneous MSI data. It also aids in the comparison of histologies across a cohort (i.e. comparison of tumor cells from one sample to another). The segmentation data for each sample in the training set is shown in the rightmost column of Figure S2 (higher magnification in Figure S3) while the battery of histological staining are in the adjacent columns.

A tile plot of average peak intensity shows univariate comparisons of 14 top marker ions for the correlated topographies (Fig. 2B). Receiver operator characteristic (ROC) curves were calculated for all picked peaks and the calculated area under the curve (a.u.c.ROC) determined the most discriminant. ROC curves measure the ability of a given signal to classify histology versus all others by determining the overlap of signal distribution between two sets of observations. In our case, observations were compared by pixels from one histology vs. a random subset of pixels from all other histologies, effectively removing markers that discriminate multiple histologies. These data reveal a selection of lipid species that are histologically discriminant and have a higher probability of being implicated in the tumor biological processes. Figure 2C shows mean picked peak data from the various histologies with the same color coding showing the peak intensity overlap for some histologies. These plots demonstrate a difficult when using single markers that are not unique in classification and the necessity of multivariate methods. Table 1 indicates the most discriminant species’ topographies, masses, a.u.c.ROCs, fold changes, identity, and characteristic MS/MS ions as identified through on tissue MS/MS (Individual ion images and annotated MS/MS spectra of the top markers are presented in Figures S4 and S5).

These data show that MSI signatures discriminate different histopathological features of CRCLM specimens. Finally, the collation of the histological and MSI data allowed us to generate a spectral library from these samples where each spectrum had a corresponding histological designation.

### Validation of Lipid signatures

**Figure 3:**
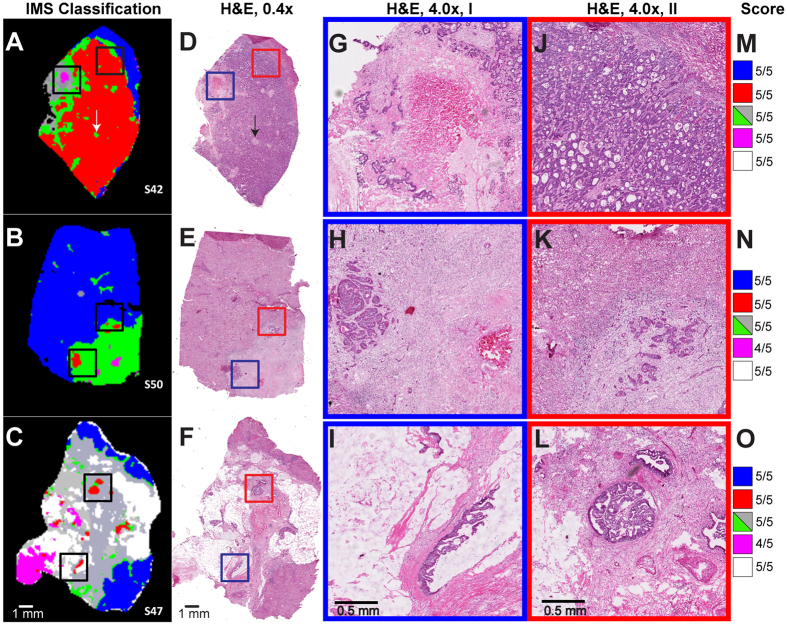
Partial Least Squares-Discriminant Analysis classifications of tissue topography (3 representative samples classified based on the previously extracted segmentations). Box (**A–C**) classified MSI image. (**D–F**) low-magnification (0.4x) H&E staining of serial section. (**G–L**) H&E staining of two areas from serial sections (high magnification, 4.0x); (**G**) enlargement of Blue box from D showing area of necrosis; (**J**) enlargement of Red Box from D showing tumor cells; (**H,I**) enlargement of Blue boxes from (**E,F**) respectively showing small foci of tumor; (**K,L**) enlargement of Red boxes from (**E,F**) respectively showing small foci of tumor; (**M–O**) Pathology correlation score. Arrows in Box (**A,D**) show small inflammation areas within tumor.

We used the spectral library developed from the training set to classify the topography of 40 additional specimens by PLS-DA. The MSI classified lipid segmentations of this cohort of specimens (Fig. 3A–C, representatives of 3 specimens) were validated by comparing a pathologist scoring of each specimen’s high resolution H&E scans (Fig. 3E–L) for the different histologies. This was done using side-by-side images of classified segmentation data and serial H&E data, where the pathologist noted the general correlation. Comparison of the MSI and pathologist’s assignment of tissue types showed strong correlation between the two (Fig. 3M–O, representing pathology score). For example, the sample in Fig. 3A MSI shows large areas of tumor cell segmentation (red coloring) indicating tumor cells verified by the H&E stain (Fig. 3D,J: magnified tumor area, tumor cells apparent by their deep purple haematoxylin stain, and in the case of differentiated CRC cells, their glandular shape). Within the dense tumor cell areas, there are small areas of tumor stroma with inflammation (Fig. 3D, indicated by arrow) evident in the H&E by their lighter pink stain, and many small monocytes, indicated as small circular cells in the staining, that are also well classified by MSI as inflammation (Fig. 3A, indicated by arrow, green segment in Fig. 3A).

Each lesion’s MSI classification was scored from 1 to 5, 1 indicating 1/5^th^ of the histology matching and 5/5^th^ complete correlation. These correlations are presented for the individual samples in the far right area of Fig. 3; panels M, N and O. MSI identified distinct lipid signatures distinguishing areas of fibrosis and inflammation. However, the pathologist was not able to evaluate the amount of inflammation if it was mixed within fibrosis (inflammatory fibrosis) on H&E staining, as further special staining is needed to differentiate between these histologies. The adjacent unaffected liver (blue), tumor (red), necrosis (purple), fibrosis/inflammation (grey/green) and loose tissue mucin (white) segments scored an average of 5, 4.9, 4.5, 4.9, and 4.7 respectively indicating a very high level of correlation between the MSI prediction of all histologies in this cohort of specimens. These correlative results based on serial sections are still useful as although many microscopic features of the tissue section change in serial sections, the macro regions change only moderately, allowing comparison at this resolution. Necrotic tissue had the lowest correlation of the major regions studied herein, and this may be due to histopathological uncertainty concerning necrotic tissue. However, the necrotic regions identified by MSI were molecularly distinct.

Classifications for a subset of our cohort (n = 22) with their validation score using the system described above are available in Figure S6 along with H&E staining of serial sections for comparison. One should note that the pathologist’s evaluation was performed visually and compared to our color schemes for each histology; therefore an absolute quantitation is not possible. Despite this reality, the correlations between MSI classification and histopathology were high.

### Mining of the necrosis segmentation identified the two distinct types of necrosis

We next evaluated whether MSI signatures can discriminate different types of necrosis in tissue specimens. In CRCLMs, two types of necrosis have been described; Infarct-Like Necrosis (ILN) and Usual Necrosis (UN). ILN is characterized by confluent areas of strong eosin staining while UN, found within tumor cell glands, also stains strongly with eosin, but is patchier and mixed with nuclear debris. Further, a ring of fibrosis sometimes surrounds ILN, whereas UN is found within the glands of colon cancer cells. ILN has been correlated with response to treatment and better prognosis, whereas UN is considered a normal process of tumor cell proliferation and is associated with a worse patient outcome^23^.

**Figure 4:**
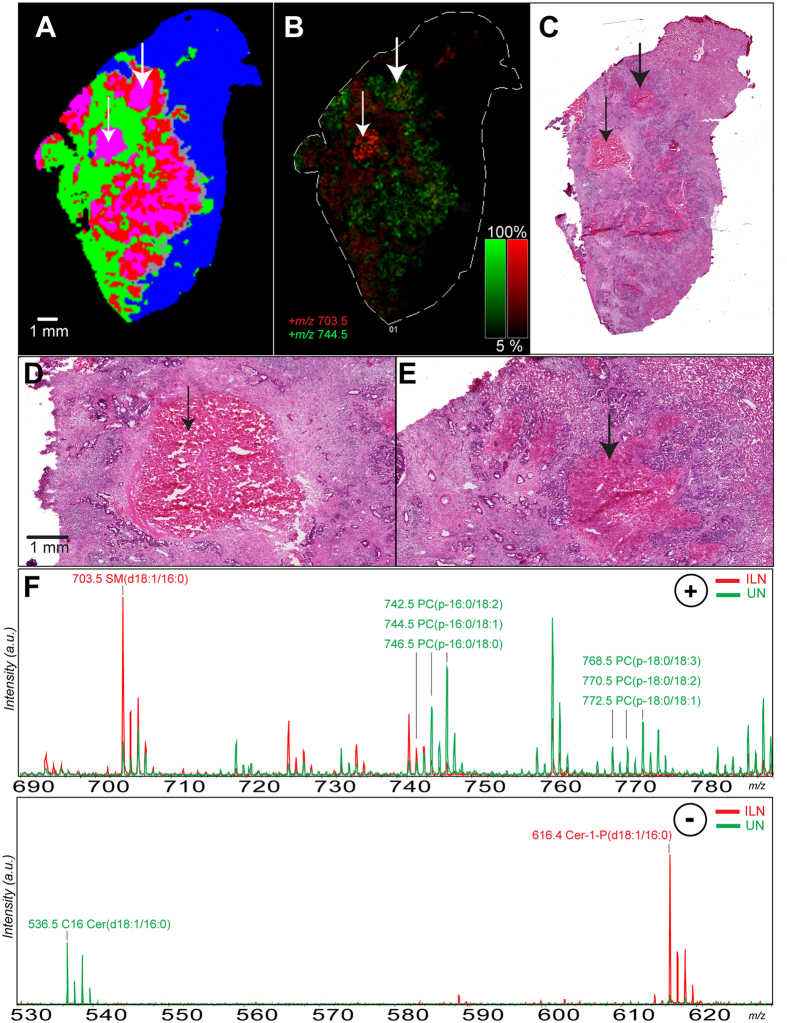
MSI detection of infarct-like necrosis (ILN) and usual necrosis (UN). (**A**) MSI classification of sample, thin left arrow in ILN, fat right arrow in UN. (**B**) Positive ionization mode ion images of *m/*z 703.57 (SM(d18:1/16:0)) in red and *m/*z 744.57 PC (p-16:0/18:1) in green. (**C**) low-magnification (0.4x) H&E staining of serial section. (**D,E)** High magnification (4.0x) of H&E staining showing areas of ILN (left) and UN (right) from serial section. (**F)** Representative spectra of the ILN and UN regions from the positive (top) and negative ionization modes (bottom), intensities are relative. Lipid species identified are described in Table 1, with MS/MS spectra available in Supplemental Figures. a.i. = arbitrary intensity.

Mining the necrosis MSI lipid signature revealed lipids that differentiated the above-mentioned types of necrosis. Figure 4A shows a classified MSI sample with a necrotic areas indicated in purple. We identified several of the species distinguishing ILN and UN in positive ionization mode with the most intense being sphingomyelin 16:0 (SM(d18:1/16:0), *m/z* 703.57), abundant in ILN (Fig. 4B, red) and several plasmalogen species (including PC(p-(16:0/18:1) and PC(p-18:0/18:3) at *m/z* 744.59 and *m/z* 765.57. Table.1, Figure S7, more abundant in UN (Fig. 4B, green). These areas were confirmed using high-magnification H&Es (Fig. 4 C–E). In negative ionization mode, we identified C16 ceramide (Cer(d18:1/16:0), *m/z* −536.50) to be abundant in UN and C16 ceramide-1-phosphate (Cer-1-P(d18:1/16:0), *m/z* −616.47) to be abundant in ILN. These species were identified through on tissue MS/MS fragmentation and their fragmentation spectra and scheme are available in Figure S8. Salient statistical information for these markers is available in Table 1.

These data demonstrate that MSI signatures can successfully discriminate different types of necrosis (ILN and UN) in tissue specimens that may not be clear by histopathology. Further, the use of both positive and negative ionization modes increased the number of discriminant species and indeed, sphingomyelin found in positive mode is related to the ceramide species in negative mode. This result hints at the fundamental tumor biology underlying necrosis by imaging ceramide directly and potential downstream or precursor species sphingomyelin and ceramide phosphates.

### Exploring clinical application/relevance of an automated recognition-based lipid-signature image analysis

**Figure 5:**
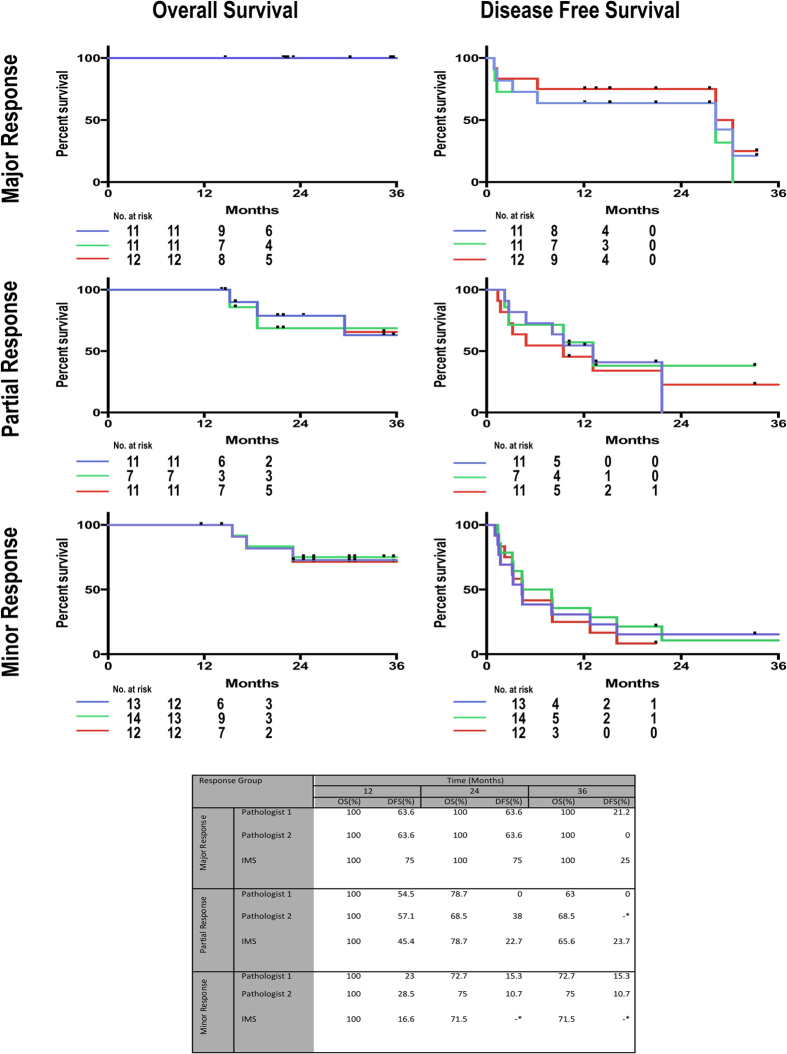
Kaplan–Meier survival analysis for the three response groups stratified according to pathological grading as observed by two independent Pathologists and by MSI. Only chemotherapy treated patients were included in survival analysis (n = 35). Pathologist 2 assessed 3 lesions to be too small for pathological evaluation. No significant differences between the three observations for each response group as assessed by Log-rank (Mantel-Cox) test. *Remaining observations censored.

To evaluate the robustness and clinical utility of MSI classifications, we calculated an mTRG score based on MSI classification data. mTRG is a CRCLM tumor grading system that scores sections stained by H&E based on the presence and amount of the following histologies: viable tumor cells, usual necrosis, infarct-like necrosis and fibrosis. We implemented the published mTRG guidelines^20^,^23^ (summarized in Table 2) into an algorithm and applied to our data. Since pathological response assessed by mTRG does not require the differentiation between the two, the MSI fibrosis (grey) and inflammation (green) segments were treated as one in the calculation of mTRG. The scores for all the specimens (n = 52) were correlated with scores of two independent, blinded pathologists (Table S2). Inter-pathologist correlation was observed at r = 0.7361 (p < 0.0001, Table 3). The MSI generated scores correlated with both pathologists (r = 0.8121 and r = 0.6227, for pathologist 1 and 2, respectively, (p < 0.0001), Table 3). To demonstrate clinical relevance, we plotted the mTRG grades from the pathologist and MSI classification to OS and DFS curves. Patients who received chemotherapy (n = 35) were stratified into three response groups according to their scores (major response: grades 1 and 2, partial response: grade 3, and minor response: grades 4 and 5^20^). Three-year OS and DFS were calculated for each group and we observed comparable outcomes in all three grading’s (Pathologist 1, Pathologist 2 and MSI lipid signatures, Fig. 5). These data confirm that MSI can be used to provide prognostically useful information with potential time–saving and automation of the entire process.

## Discussion

A challenge in the treatment of patients is the assessment of their response in an objective and quantitative manner^39^. Currently a clinician relies on both the pathologist and radiologist’s assessments, which remain subjective and differs based on experience. Pathologist assessments rely on “eye-ball” estimates of tumor burden and with the introduction of mTRG (differentiating the two types of necrosis) its subjectivity has been driven further. Working towards more objective patient assessments, we have succeeded in converting the mTRG from a subjective semi-quantitative grading system into a computational model that calculates a mTRG score from numerical MSI data. The mTRG calculated from MSI data was compared to the pathologist’s grading in order to stratify patients according to their OS and DFS and found to have a strong correlation and good predictive power. By introducing a computational method based on numeric MSI data we start to unify patient response grading, and move towards a well-defined synoptic reporting tool.

Our approach to data treatment relies on histopathology correlated MSI segmentation to define our classification groups and is advantageous when compared to histology driven MSI approaches. The latter selects ROIs for MSI analysis based on outside annotation post-MSI, ignoring unannotated pixels, where our approach includes the entirety of the MSI data available in the analysis, reducing error and increasing statistical power for inter-sample comparison. Although similar approaches have been described before to distinguish cancer lesions from adjacent normal tissue using lipid signal intensities, previous studies have not partitioned the cancer lesions into their component histologies in the depth described here^33^,^40^. The inclusion of all of the regions is beneficial as it describes the tumor burden and the tumor microenvironment. Assessing the whole lesion is important because cancer lesions are composed of multiple tissue types and cancer progression and regression is a dynamic process with tissue composition changes reflecting the status of the lesion.

The clinical application of MSI lipid signatures goes beyond pathological grading as it can serve as a valuable tool for refining current grading parameters and the discovery of candidate biomarkers. For example, within the areas of necrosis we identified C16 ceramide and C16 C1P as biomarkers of UN and ILN, respectively. C16 ceramide has been described as having tumor suppressor activity (having both anti-proliferative and pro-apoptotic activities) while in contrast C1P has pro-survival and anti-apoptotic activity, making these lipids attractive biomarker candidates for response to therapy^41^,^42^. It has been reported that high levels of C1P, which stimulates cell division and inhibits apoptosis, is toxic and can kill cells^43^. Alongside the ceramides, plasmalogens were identified in the necrosis areas. Although found ubiquitously in human cells, they have not been previously described in cancer or necrosis processes, unlike other ether lipids. In addition, they have been reported to be lower in abundance in liver tissue compared to other organs^43^,^44^. Interestingly, within our data set we identified PE (phosphoethanolamine) plasmalogens to be associated with both tumor areas and areas of inflammation, whereas PC plasmalogens are exclusively abundant in areas of UN. Current research indicates PE plasmalogens as the precursor to PC plasmalogens as no plasmenylcholine desaturase enzyme has been described. Compared to UN, we find minimal PC plasmalogen signal in surrounding liver tissue. The function of these lipids in the specific histologies opens further areas of research. The ability to distinguish between UN and ILN is extremely important and not always obvious when examining CRCLMs histologically. We have identified unique lipid biomarkers for each of these tissue types. In fact we are able to identify ILN areas within UN that has been graded by pathologists to be entirely UN. These findings warrant investigation in a larger series of patients to determine if it impacts prognosis or response to therapeutic interventions as well as to describe the relationship between all of the lipids present in the two types of necrosis.

From a technical standpoint, classification based on MSI data will be key in clinical analysis. It will not be practical for a routine clinical pathology lab to examine MSI data ion by ion. Furthermore, many molecules detected by MSI are generic and will be expressed by several types of cells, with changes in their relative abundance distinguishing histologies. By building a larger more heterogeneous spectral library with various types of cancers MSI could be used for diagnostic purposes in differentiating cancer type based on lipid profiles. Although the basis of this study was MSI data of lipids, lipids can be analysed by other MS systems, most notably LC-MS. LC-MS is not yet suitable for high throughput spatially resolved analysis of tissue lipids, but provides a deeper coverage of lipids. Surface extraction of lipids from tissue into an LC-MS may give the depth of lipidome coverage to fully map the metabolomic pathways in tumors previously only hinted at by MSI.

This study supports an evidence-based model for decision making in regards to diagnosis, prognosis and intervention for CRCLM. We demonstrate functional linkages between MSI and histopathology and the utility of this “omics” technology in the clinical setting as a companion diagnostic and prognostic tool for pathologists and clinicians. Finally, using lipidomics we have uncovered a novel set of markers (ceramides and plasmalogens) to start investigating the mechanism of treatment effects and possibly novel drug targets. The introduction of MSI into the clinic and as a new tool for biomarker discovery will dramatically change the field of molecular pathology.

## Methods

### Standard Protocol Approvals, Registrations, and Patient Consents

The study was done in accordance with the guidelines approved by McGill University Health Centre Institutional Review Board (IRB). A prior written informed consent was obtained from all the subjects to participate in the study.

#### Clinical Data

This retrospective study included a total of 52 lesions from 50 patients. Resections were preformed between November 2011 and July 2014. Clinical data was collected for each patient through the locally established hospital database and medical records. Included within the data are demographics, primary and metastatic disease characteristics, relevant laboratory results, chemotherapy and co-morbidities. As shown in the supplemental Table S3, median age of diagnosis was 63 (range 31–81) years. Rectal cancer accounted for 34% of the cases. Approximately two thirds (64%) of the patients had synchronous liver metastasis (developed metastasis within a year of diagnosing the primary). Seventeen lesions were chemo-naive while the rest received neoadjuvant chemotherapy with an average of 7 cycles (Range 3–28). Estimated 1 and 3-year OS is 100% and 82.6% respectively. Twenty-seven (54%) of patients had recurrence in the liver, estimated 1-year and 3-year DFS is 49.9% and 44.4% respectively (26.5 months mean follow up duration).

#### Tissue sample acquisition

Informed consent was obtained from all patients through the McGill University Health Centre (MUHC) Liver Disease Biobank (LDB: MUHC research ethics board approved protocol). Surgical specimens were procured and released to the Biobank immediately after the pathologist’s confirmation of carcinoma and surgical margins. The specimens were frozen, within 30 minutes, according to the LDB standard operating procedures and processed as previously described^45^.

#### Histochemical Staining

Frozen tissues were cut using a cryostat at 10 μm, and stored at −80 °C in tightly closed boxes until staining. Before use, the slides were allowed to dry on the benchtop for 10 minutes at room temperature and then fixed for 60 minutes in freshly made 4% formaldehyde dissolved in 0.1 M phosphate buffer at pH 7.2. Comparative topography assays were performed between MSI and different histological stains: Apolipoprotein F (ApoF), ISH, PLTP ISH, alcian blue (AB)^46^, H&E, and Ki67 immuno-staining^47^, of the training set. ApoF ISH is hepatocyte-specific, defining regions of liver adjacent to the tumor area; PLTP ISH labeling revealed the concentration of macrophages within or around the tumor; AB stains areas of the metastatic lesions that contain mucin; H&E staining provided basic histological information for MSI correlation and pathological grading; Ki67 immuno-staining identified tumor cells with cell cycle activity.

#### *In situ* hybridization

*In situ* hybridization (ISH) was performed with [^35^S]-labeled riboprobes synthesized *in vitro* from DNA Templates. Briefly, mouse Apolipoprotein F (ApoF, GenBank AF411832.1) DNA template of 675 bp was produced by PCR using sense gataccagatgcagacctca and antisense gttcgtcgttgttgacaaga primers. Human phospholipid transfer protein (PLTP, GenBank NM_006227.3) DNA Template of 884 bp was produced using GAAGAGCGGATGGTGTATGT (sense) and TGGTGGACGGACTGTAATTG (antisense) primers. Sequences recognized by SP6 Polymerase (5′-GCATTAATTTAGGTGACACTATAGAAGCG-3′) were attached to antisense and T7 Polymerase (5′-GCGCTATAATACGACTCACTATAGGGAGA-3′) to sense primers. Following hybridization, the results were visualized by x-ray film autoradiography showing anatomical level topography and emulsion autoradiography showing cellular level ISH labeling. Figure S2 represents the staining of the training set lesions.

#### Pathological Evaluation Methods

The following methods were used and compared to evaluate our lipid signatures:

- Modified tumor regression grading (mTRG): All 52 lesions were stained (detailed in Supplement Figure S2) to identify areas and relative percentage of viable liver cells, fibrosis, inflammation, mucin, usual necrosis and infarct like necrosis. Two independent pathologists assessed modified Tumor Regression Grade (mTRG) as described by *Chang et al*. for every sample using high-resolution H&E slide scans (Table S2)^23^. For the mTRG grading UN was defined as containing nuclear debris and bordered by viable cells whereas ILN was defined as being a large confluent areas of eosinophilic cytoplasmic remnant located centrally within the lesion without the presence of nuclear debris. Briefly, mTRG1 is defined by the absence of tumor cells and replaced by fibrosis and ILN; mTRG2 contains rare scattered residual tumor cells with predominant fibrosis and the presence of both ILN and UN; mTRG3 contains more residual tumor cells throughout predominant fibrosis and UN; mTRG4 contains large amounts of tumor cells and intermingled UN which predominates over fibrosis and ILN; and mTRG5 contains tumor cells and intermingles UN without any fibrosis. The higher the grade the worse the response.
- Pathological response: All samples were grouped into 3 response groups based on their mTRG score. Major response includes: grades 1 and 2, partial response: grade 3, and minor response: grades 4 and 5^20^.

#### Statistical Analysis

Survival probabilities were calculated by the Kaplan-Meier method and compared by the log rank (Mantel-Cox) test. A p value of less than 0.05 was considered statistically significant. All analysis was performed using JMP.11 software. Overall survival was calculated from the date that the metastases were detected to the date of last follow-up. Disease free survival was calculated from the date of surgical intervention to the date of recurrence or last follow-up if patient was still disease free.

#### Mass Spectrometry

##### MALDI-MSI Tissue Sectioning and Sample Preparation

Tissue sectioning and preparation was performed as previously described^45^. Briefly, tissue cryo-sections of 10 μm were thaw-mounted onto ITO coated glass slides (Delta Technologies, Loveland, CO) and 1,5-diaminonapthalene (DAN) matrix (Sigma Aldrich, Oakville, CA) was sublimated on the slide.

##### MALD-MSI instrument parameters

MSI in both positive and negative polarity of each tissue section was performed using a Bruker MALDI-TOF/TOF Ultraflextreme as previously described with only minor changes^45^. Briefly, 150 laser shots were summed per array position, with 100 μm of resolution for every acquisition. Negative ionization mode was acquired using an offset of 50 μm to positive ionization mode MSI grid array. MALDI laser influence and ion accelerating voltages in reflectron mode were optimized for the sample set. The mass range was *m*/*z* 460–1200 for both ionization modes.

##### On tissue MALDI-MS/MS

MS/MS measurements for species detected in positive mode were acquired in LIFT-TOF/TOF mode of the Ultraflexetreme and with a Bruker Solarix 15 T FT-ICR using dried droplet spotting of 2,5-dihydroxyacetophenone doped with 100 mM Lithium Trifluoroacetate for improved fragmentation^48^ whereas negative species were directly fragmented after DAN sublimation. MS/MS data were processed using flexAnalysis v3.3 software (Bruker Daltonics, Billerica, MA). The LIPID MAPS database was used for comparing accurate mass and obtaining lipid structures for the determination of fragmentation pathways^49^.

##### MALDI-MSI data analysis

Data was displayed using flexImaging 4.1 (Bruker Daltonics, Billerica, Massachusetts). Data was exported to imzML format for processing^50^. Spectral smoothing using the Savitzky-Golasky algorithm, Total Ion Current (TIC) normalization, and peak picking using a signal-to-noise ratio of 3.0 were performed using the MALDIquant package in the R environment^51^. Generated peak intensity data were segmented using spatially aware K-means (r = 1, k = 7)^52^, as implemented in the Cardinal MSI R package^53^. Partial Least Squares–Discriminant Analysis (PLS-DA) and ROC analysis were performed in the R environment using the mixOmics^54^ and ROCR^55^ packages, respectively.

## Additional Information

**How to cite this article**: Patterson, N. H. *et al*. Assessment of pathological response to therapy using lipid mass spectrometry imaging. *Sci. Rep*. **6**, 36814; doi: 10.1038/srep36814 (2016).

**Publisher’s note:** Springer Nature remains neutral with regard to jurisdictional claims in published maps and institutional affiliations.

## Supplementary Material

Supplementary Information

## Figures and Tables

**Table 1:** Table of characteristic ions.

Topography	*m/z*	Identity	Fold change	a.u.c.ROC	MS/MS
Normal	NEG_738.5	PE(16:0/20:4)	3.161 ± 0.23	0.923	FA-1:255, FA-2:303, 434(Etn), 452(Etn)
	NEG_762.5	PE(16:0/22:6)	4.827 ± 0.45	0.940	FA-1:255, FA-2:327, 434(Etn), 452(Etn), 506(Etn), 524(Etn)
	NEG_885.56	PI(18:0/20:4)	3.227 ± 0.19	0.964	303, 283 (fatty acyl chains), 297 (Glycerophosphoinositol), 241 (Inositol phosphate ion)
	POS_758.57	PC(16:0/18:2)	2.71 ± 0.66	0.996	Li_fragmentation: 508(NL of 16:0), 484(NL of FA 18:2), 508(NL of 16:0), NL of 59, NL of 183 (PC headgroup)
Tumor	NEG_698.48	PE(p-16:0/18:2)	2.408 ± 0.42	0.878	FA-2: 279, 436 (Loss of sn2 acyl chain as ketene (RCH = C = O) from [M-H]-)
	NEG_700.51	PE(p-16:0/18:1)	2.151 ± 0.52	0.864	FA-2: 281, 436 (Loss of sn2 acyl chain as ketene (RCH = C = O) from [M-H]-)
	NEG_835.54	PI(16:0/18:1)	2.813 ± 0.63	0.885	281, 255 (fatty acyl chains), 297 (Glycerophosphoinositol), 241 (Inositol phosphate ion), 673(NL of inositol)
	POS_706.55	PC(14:0/16:0)	2.835 ± 0.51	0.829	184(PC headgroup), ~0.7 ppm error
	POS_732.55	PC(16:0/16:1)	5.355 ± 1.29	0.921	Li_fragmentation: 480(NL of 16:1), 482(NL of 16:0), NL of 59, NL of 183 (PC headgroup)
Inflammation	NEG_722.49	PE(p-16:0/20:4)	3.749 ± 0.62	0.935	436(Loss of sn2 acyl chain as ketene (RCH = C = O) from [M-H]-), 303(FA chain)
	NEG_750.53	PE(p-18:0/20:4)	3.744 ± 0.66	0.845	464 (Loss sn2 acyl chain as ketene (RCH = C = O) from [M-H]-), 303(FA)
	POS_734.57	PC(16:0/16:0)	3.673 ± 0.72	0.942	Li_fragmentation: 478(NL of 16:0), NL of 59, NL of 183 (PC headgroup)
Fibrosis	POS_782.55	PC(18:2/18:2)	1.5 ± 0.61	0.780	184(phosphatidylcholine headgroup)
Infarct-like Necrosis	POS_703.57	SM(d18:1/16:0)	5.62 ± 0.66	0.902	Li_fragmentation: 280(sn-2 loss), NL of 59, NL of 183 (PC headgroup)
	NEG_616.47	Cer-1-P(d18:1/16:0)	4.83 ± 1.12	0.880	96(phosphate group), 78(phosphate-H_2_O)
Usual Necrosis	POS_742.57	PC(p-16:0/18:2)	2.20 ± 0.43	0.823	Li_fragmentation: NL of 189 (PC headgroup), 279(NL of 189 + NL of non-plasmenyl FA)
	POS_744.59	PC(p-16:0/18:1)	5.16 ± 1.14	0.911	Li_fragmentation: NL of 189 (PC headgroup), 279(NL of 189 + NL of non-plasmenyl FA)
	POS_746.59	PC(p-16:0/18:0)	5.56 ± 0.78	0.912	Li_fragmentation: NL of 189 (PC headgroup), 279(NL of 189 + NL of non-plasmenyl FA)
	POS_768.57	PC(p-18:0/18:3)	5.02 ± 0.98	0.902	Li_fragmentation: NL of 189 (PC headgroup), 307(NL of 189 + NL of non-plasmenyl FA)
	POS_770.59	PC(p-18:0/18:2)	3.94 ± 0.74	0.872	Li_fragmentation: NL of 189 (PC headgroup), 307(NL of 189 + NL of non-plasmenyl FA)
	POS_772.59	PC(p-18:0/18:1)	3.54 ± 0.88	0.854	Li_fragmentation: NL of 189 (PC headgroup), 307(NL of 189 + NL of non-plasmenyl FA)
	NEG_536.50	C16 Cer(d18:1/16:0)	4.83 ± 1.19	0.945	506(NL of H_2_CO), 504(NL of H_2_-H_2_CO), 488(NL of H_2_O-H_2_CO), 296(side chain loss), 254(FA loss)

Fold change and area under the ROC calculated vs all other histologies. Etn = ethanolamine. NL = neutral loss. FA = fatty acid. PC = phosphatidylcholine. PE = phosphoethanolamine. SM = Sphingomyelin. p-16:0/p-18:0 = plasmenyl lipid. Cer = Ceramide. PI = phosphoinositol.

**Table 2:** Algorithm used for mTRG Published grading guidelines used for scoring all specimens (n = 52).

mTRG	MSI lipid signature profile	
	Area viable tumor + UN (%)	Area fibrosis + inflammation (%)
1	0	0–100
2	<7*	0–100
3	≥7 and <50	≥50
4	≥50	≥5
5	≥50	0–5

*7% was used as cutoff point to correlate with ‘rare’ tumor foci by a clinical pathologist.

**Table 3:** Correlation of pathologist and MSI mTRG (n = 52).

	Pathologist 1	Pathologist 2	MALDI MSI
Pathologist 1		0.7361	0.8121
Pathologist 2	0.7361		0.6227
MALDI MSI	0.8121	0.6227	
Average	0.7741	0.6794	0.7174

Showing a high overall correlation of MSI based grading scores with the two pathologists (r = 0.717, p < 0.0001).

P < 0.0001 for all correlations.
