# Supplementary material for: Assessment of pathological response to therapy using lipid mass spectrometry imaging
Source: Sci Rep. 2016 Nov 14;6:36814. doi: 10.1038/srep36814 (PMC5107952; doi:10.1038/srep36814)
Supplement: Supplementary Information [file srep36814-s1.pdf]

Title: Assessment of pathological response to therapy using lipid mass spectrometry imaging

Nathan Heath Patterson[1*], Balqis Alabdulkarim[2,3*], Anthoula Lazaris[2,3], Aurélien Thomas[4,5], Martin M. Marcinkiewicz[6], Zu-hua Gao[3,7], Peter B. Vermeulen[8], Pierre Chaurand[1$] and Peter Metrakos[2,3$]

1) Department of Chemistry, University of Montreal, Montreal, Quebec, Canada

2) Department of Surgery, McGill University Health Center, Quebec, Canada

3) Cancer Research Program, McGill University Health Center-Research Institute

4) Unit of Toxicology, University Centre of Legal Medicine, Geneva-Lausanne, Switzerland

5) Faculty of Biology and Medicine, Lausanne University Hospital, University of Lausanne, Lausanne, Switzerland

6) Cytochem Inc., 6465 Durocher Avenue, Montreal, Quebec, Canada

7) Department of Pathology, McGill University Health Center, Quebec, Canada

8) Translational Cancer Research Unit, GZA Hospitals St.-Augustinus, 2610, Wilrijk, Belgium

* These authors contributed equally

$ Corresponding authors:

Peter Metrakos<br><br>Department of Surgery, Section of Hepato-biliary and Transplant Surgery,<br>McGill University Health Center,<br>Montreal (Quebec)<br>Canada	Tel: (+1) 514 843 1600<br>Fax: (+1) (514) 843-1434<br>E-mail: peter.metrakos@mcgill.ca
Pierre Chaurand<br><br>Department of Chemistry<br>Université de Montréal<br>Montreal (Quebec)<br>Canada	Tel: (+1) 514 343 2088<br>Fax: (+1) 514 343 7586<br>E-mail: pierre.chaurand@umontreal.ca

Supplemental Table 1. Patient characteristics separated by training and validation set. Training set 12 lesions from 12 patients. Validation set 40 lesions from 38 patients (2 patients underwent stage resection). *n=38 **n=40

**Variable**	**Training Set**			**Validation set**		
	**No.(%)**	**Mean (SD)**	**Range**	**No.(%)**	**Mean (SD)**	**Range**
Age (years)		62.7 (15.9)	31-81		*63.2 (11.7)	*40-81
Sex						
Male	5 (41.7)			*26 (68.4)		
Female	7 (58.3)			*12 (31.5)		
Location of Primary Disease						
Proper Colon	4 (33.3)			*11(28.9)		
Sigmoid Colon	4 (33.3)			*5 (13.15)		
Rectum	4 (33.3)			*14 (36.8)		
Unknown	0 (0)			*8 (21)		
Stage of Primary Disease (TNM)						
T (size)						
T1	0 (0)			*1 (2.6)		
T2	0 (0)			*2 (5.2)		
T3	7 (58.3)			*20 (52.6)		
T4	4 (33.3)			*4 (10.4)		
Unknown	1 (8.3)			*11 (28.9)		
N (lymph node involvement)						
N0	3 (25)			*8 (21)		
N1	6 (50)			*10 (26.3)		
N2	2 (16.6)			*7 (18.4)		
Unknown	1 (8.3)			*13 (34.2)		
Disease Free intevral						
Synchronous	12 (100)			**21 (52.5)		
Metachrnous	0 (0)			**19 (47.5)		
Location of Liver Lesion						
Right Lobe	9 (75)			**20 (50)		
Lift Lobe	3 (25)			**18 (45)		
Bilobar	0 (0)			**2 (5)		
No. of Lesions		2.1 (1.9)	1-7		**2.6 (1.9)	**1-10
≤ 4	10 (83.3)			**29 (72.5)		
> 4	2 (16.6)			**11 (27.5)		
Size of Lesion (cm)		4.7 (2.3)	0.3-9.2		**3.4 (2.3)	**0.8-10
≤ 5 cm	6 (50)			••31(77.5)		
> 5 cm	6 (50)			••9 (22.5)		
Chemotherapy Status						
Chemo-naive	3 (25)			••14 (35)		
Treated	9 (75)			••26 (65)		

Supplemental Table 2: Pathologists and MSI mTRG gradings for all samples.

serial no.	Path1	Path2	MSI
1	1	2	1
2	2	2	2
3	3	3	3
4	3	5	3
5	3	3	3
6	4	4	4
7	4	5	3
8	2	2	1
9	4	4	4
10	4	5	4
11	5	4	4
12	3	4	3
13	3	3	3
14	3	too small to assess	3
15	4	5	3
16	3	too small to assess	3
17	4	5	3
18	4	4	4
19	1	too small to assess	1
20	4	3	3
21	4	4	3
22	2	1	1
23	4	4	4
24	2	1	2
25	3	5	4
26	3	3	4
27	2	too small to assess	3
28	2	2	2
29	3	2	2
30	4	4	3
31	3	3	3
32	4	too small to assess	4
33	3	3	3
34	1	1	1
35	3	2	3
36	3	3	3
37	4	4	4
38	4	4	4
39	4	1	5
40	3	3	3
41	4	4	3
42	4	4	4
43	4	4	3
44	4	4	4
45	4	4	3
46	4	4	4
47	2	2	2
48	3	4	4
49	4	5	4
50	2	2	2
51	3	3	2
52	4	4	4

Supplemental Table 3. Patient characteristics for the entire population, *n=50 **n=52

Variable	No.(%)	Mean (SD)	Range
Age (years)*		63.1 (11.7)	31-81
Sex*			
Male	31 (62)		
Female	19 (38)		
Location of Primary Disease*			
Proper Colon	8 (16)		
Sigmoid Colon	15 (30)		
Rectum	17 (34)		
Unknown	10 (20)		
Stage of Primary Disease (TNM)*			
T (size)			
T1	1 (2)		
T2	2 (4)		
T3	27 (54)		
T4	8 (16)		
Unknown	12 (24)		
N (lymph node)			
N0	11 (22)		
N1	16 (32)		
N2	9 (18)		
Unknown	14 (28)		
Disease Free interval**			
Synchronous	33 (63.4)		
Metachronous	19 (36.5)		
Location of Liver Lesion**			
Right Lobe	31 (59.6)		
Lift Lobe	19 (36.5)		
Bi-lobular	2 (3.8)		
No. of Lesions**		2.5 (2.1)	1-10
≤ 4	43 (82.6)		
> 4	9 (17.3)		
Size of Lesion (cm)**		3.7 (2.3)	0.3-10
≤ 5 cm	36 (69.2)		
> 5 cm	16 (30.7)		
**Chemotherapy Status**			
Chemo-naive	17(32.6)		
Chemo-treated	35(67.3)		

Supplemental Figure 1. Overall Survival (OS) and Disease Free Survival (DFS). A Training Set: estimated 1-year and 3-year OS were observed at 100% and 81.8% respectively. Estimated 1-year and 3-year DFS were observed at 58.3% and 48.6% respectively. **B** Validation set: estimated 1-year and 3-year OS were observed at 100% and 81.6% respectively. Estimated 1-year and 3-year DFS were observed at 47.3% and 44.2% respectively.

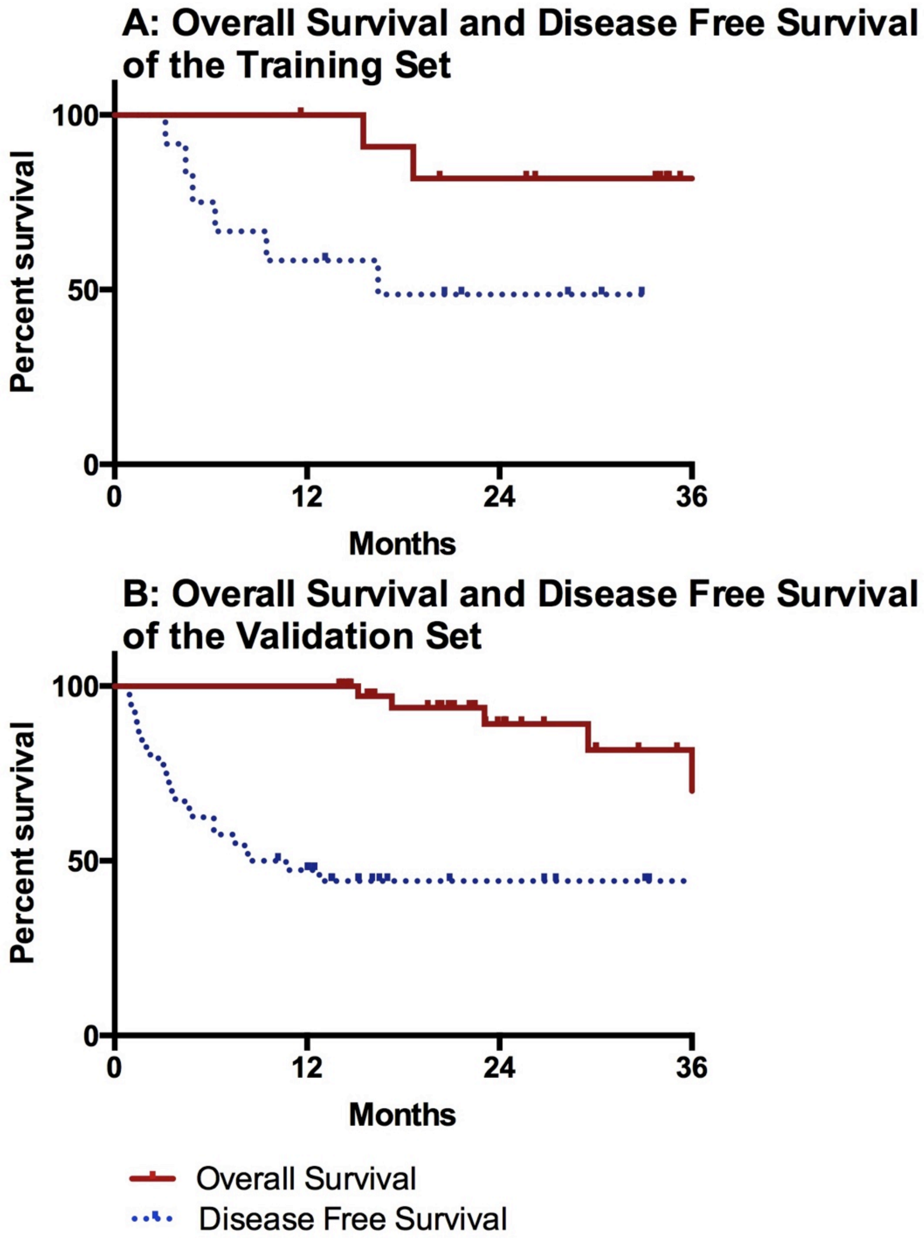

Supplemental Figure 2:

Comparative topography of training set representative CRCLM resections. Column 1: x-ray film auto-radiography showing ApoF mRNA hepatocyte-specific in situ hybridization labeling. Column 2: Alcian Blue (AB) staining pattern in tumor tissue. Column 3: tissue topography with H&E staining. Column 4: x-ray film auto-radiography with macrophage-preferential PLTP mRNA in situ hybridization labeling. Column 5: ki-67 immunostaining indicating viable tumor cells. Column 6: Hyper-spectral spatially aware *k-means* clustering mass spectrometry imaging (MSI) data. Abbreviations: H – hepatocyte; Li – liver; M – macrophage; S – selection and T – tumor. Magnification x5.

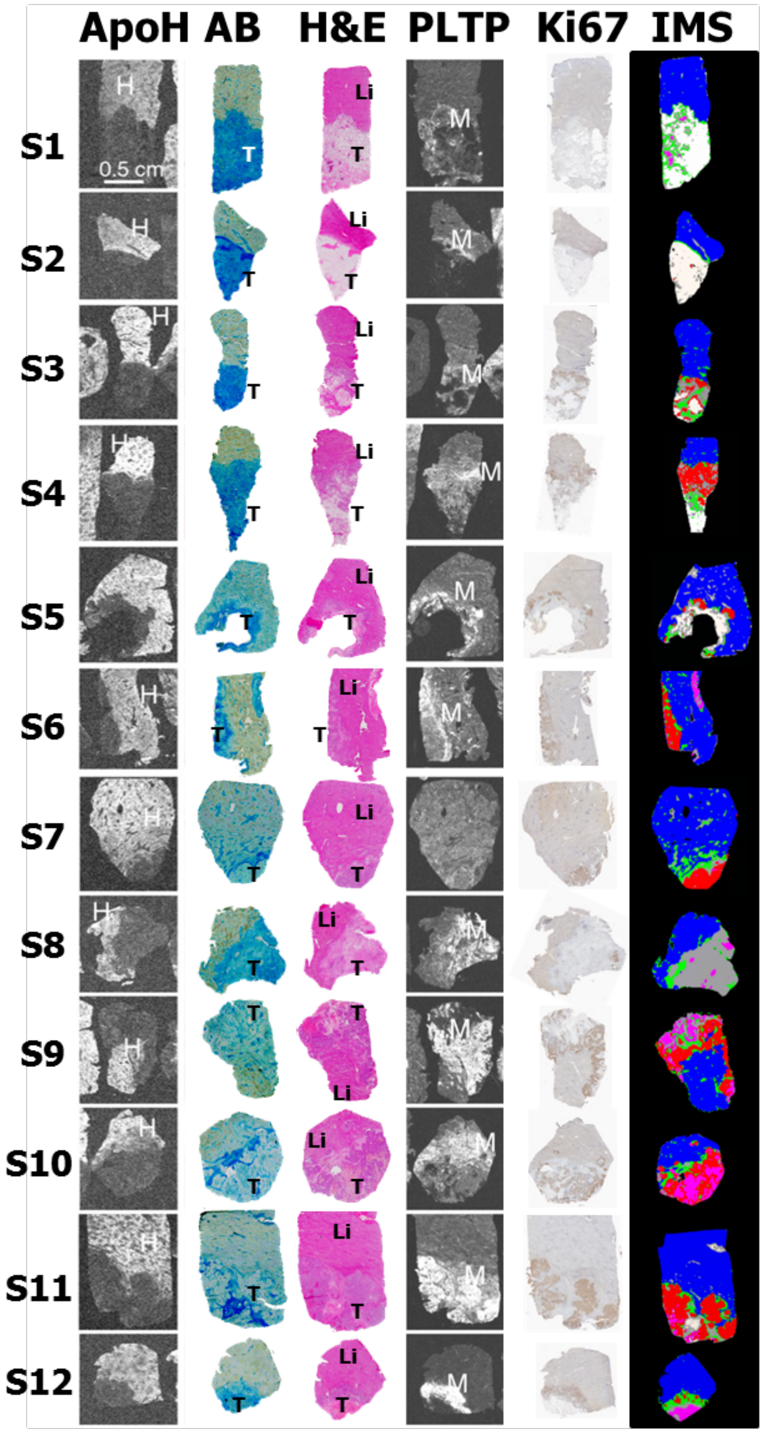

Supplemental Figure 3: Segmentation of the MSI data for the training set. The colors blue, red, purple, green, grey and white represent normal liver, viable tumor, necrosis, inflammation, fibrosis, and mucinous/empty/noise areas in and outside the tumor area, respectively

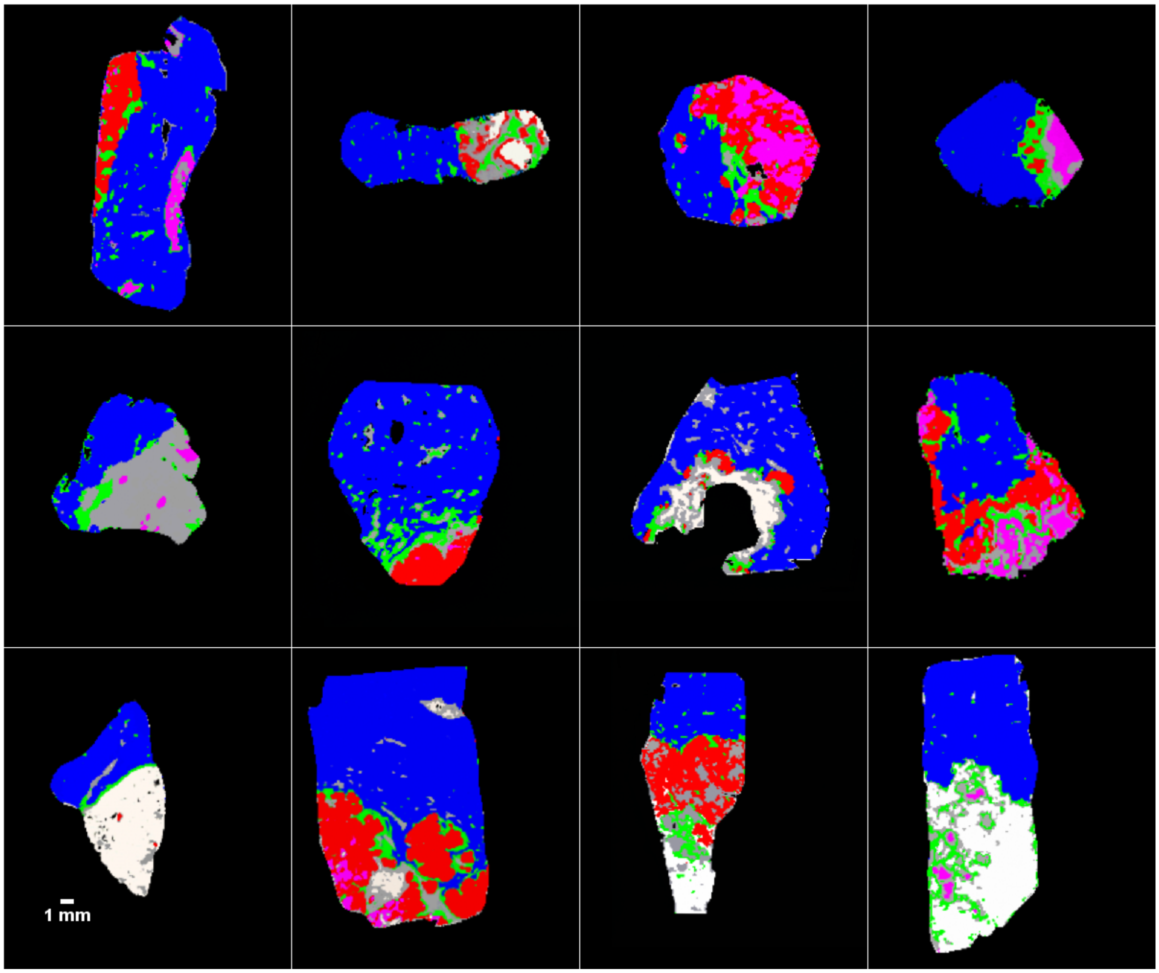

Supplemental Figure 4:

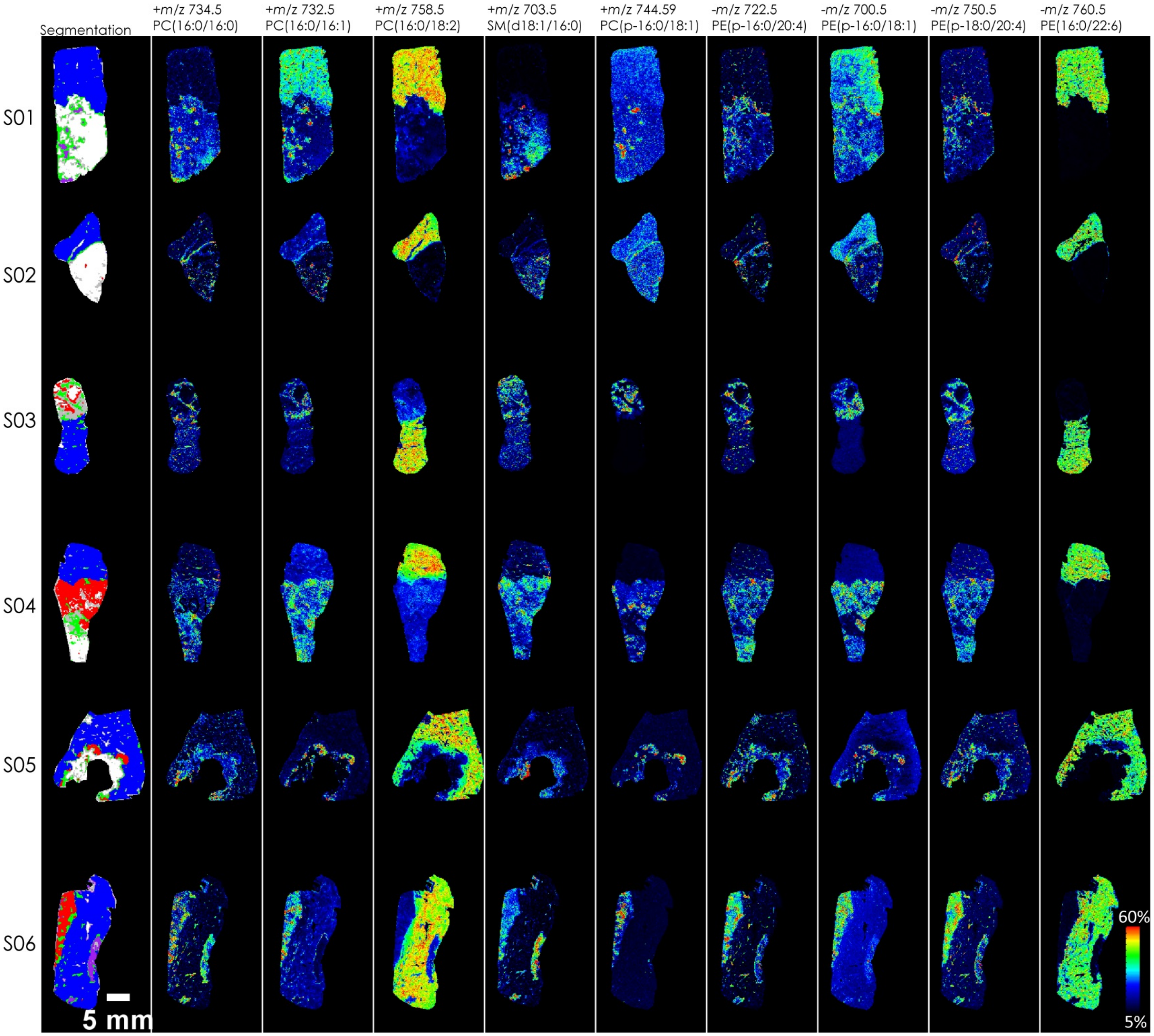

Subset of ion images of discriminant ions in training set.

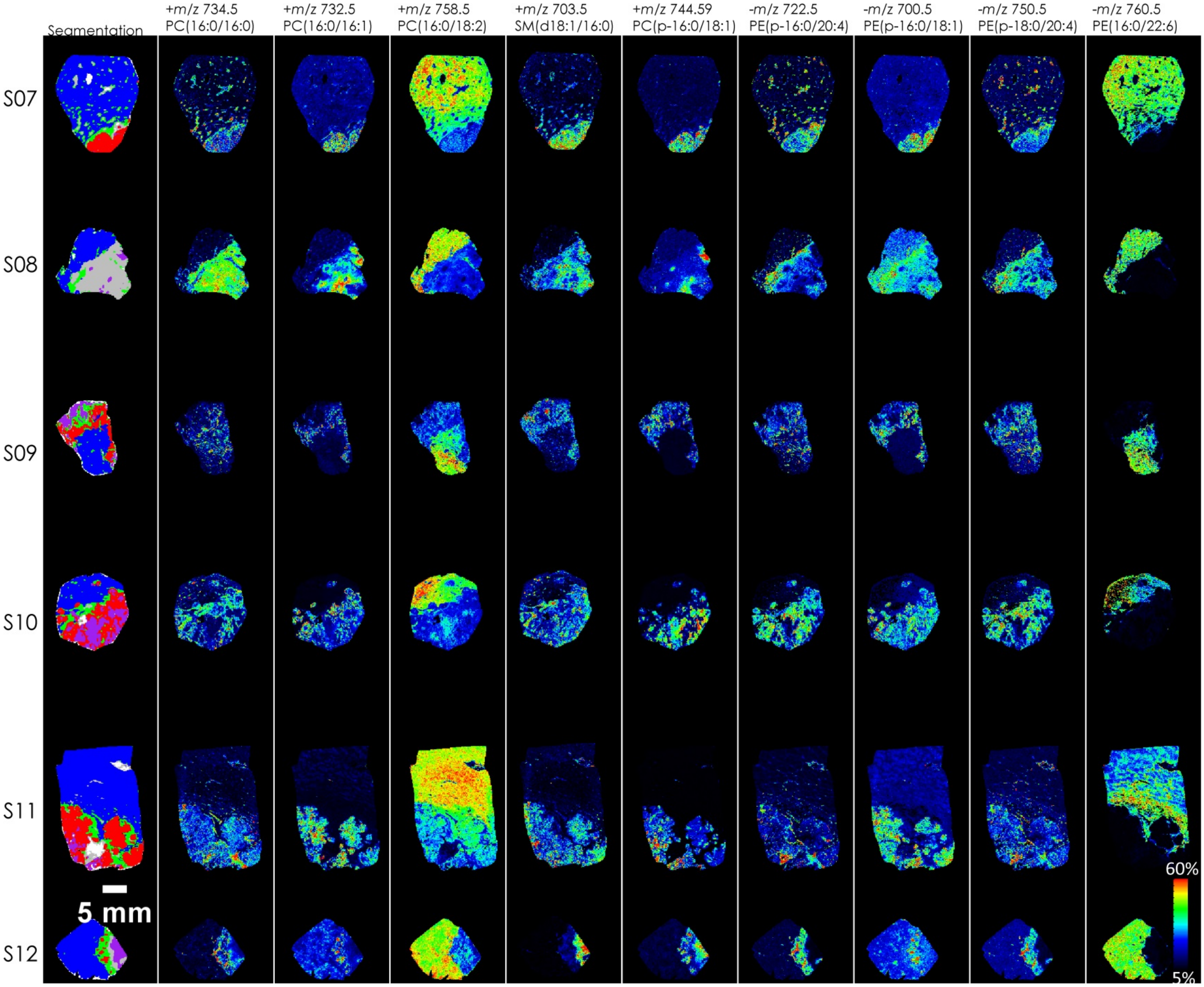

Subset of ion images of discriminant ions in training set.

Supplemental Figure 5:

MS/MS of most discrimiant markers. Fatty acid side chain length and number of unsaturations are given, however, side chain position at either *sn-1* or *sn-2* is not known.

Histology, ionization mode, theoretical mass, ion form, species

I. Adjacent "Normal" Liver, Negative mode, *m/z* 738.62 [M-H]-, PE(16:0/20:4)

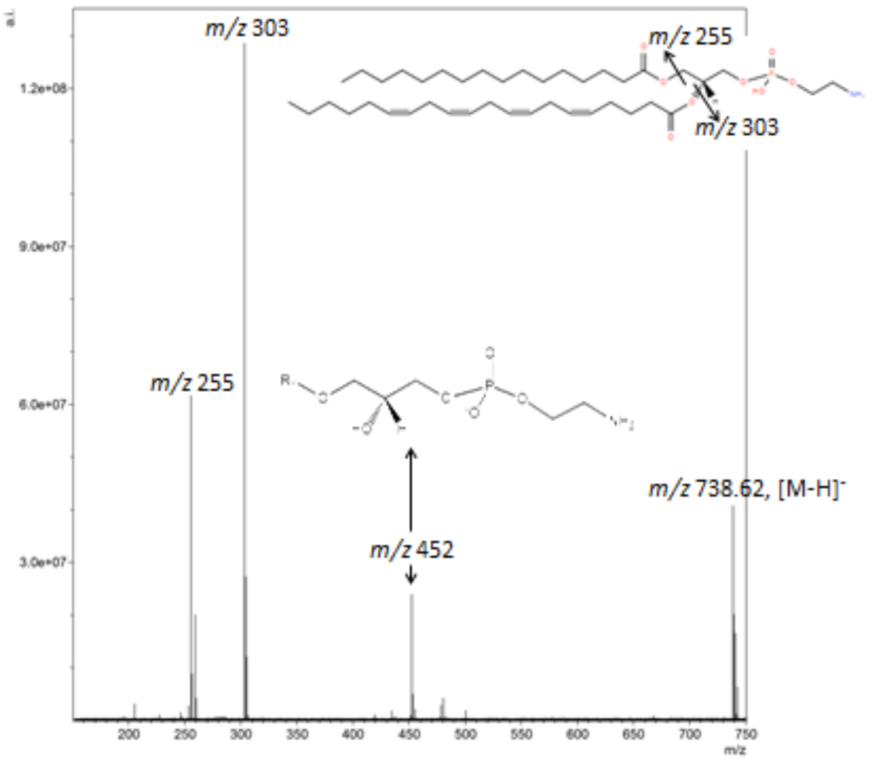

II. Adjacent "Normal" Liver, Negative mode, *m/z* 762.55 [M-H]-, PE(16:0/20:4)

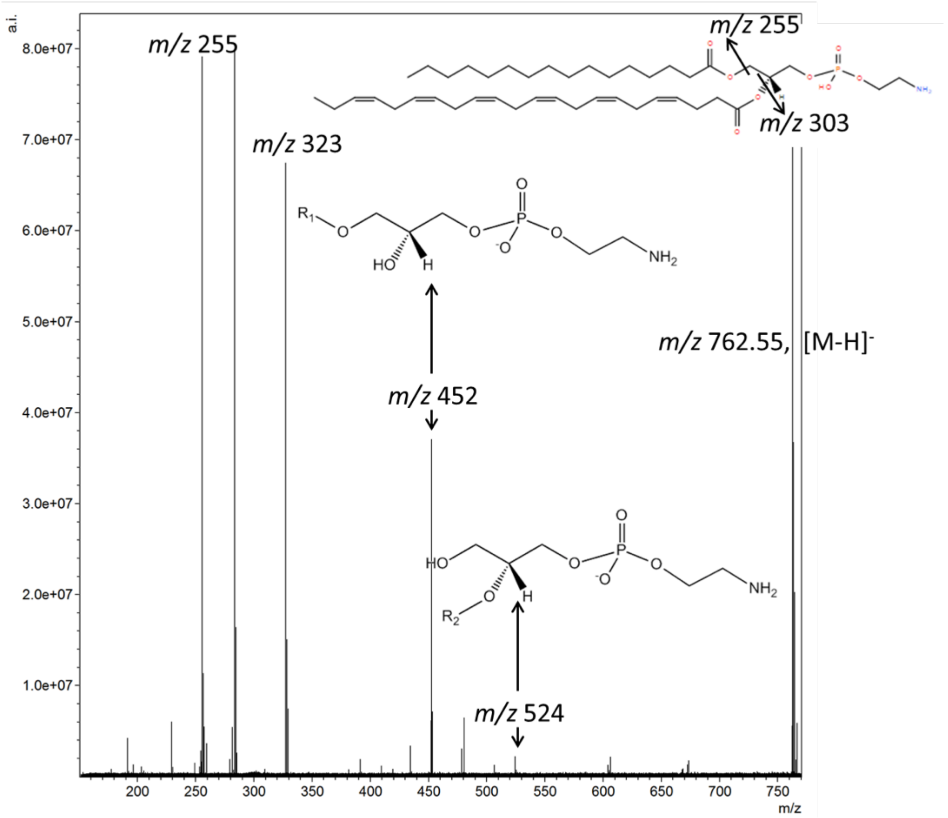

III. Adjacent "Normal" Liver, Negative mode, *m/z* 885.56 $[M-H]^-$, PI(18:0/20:4)

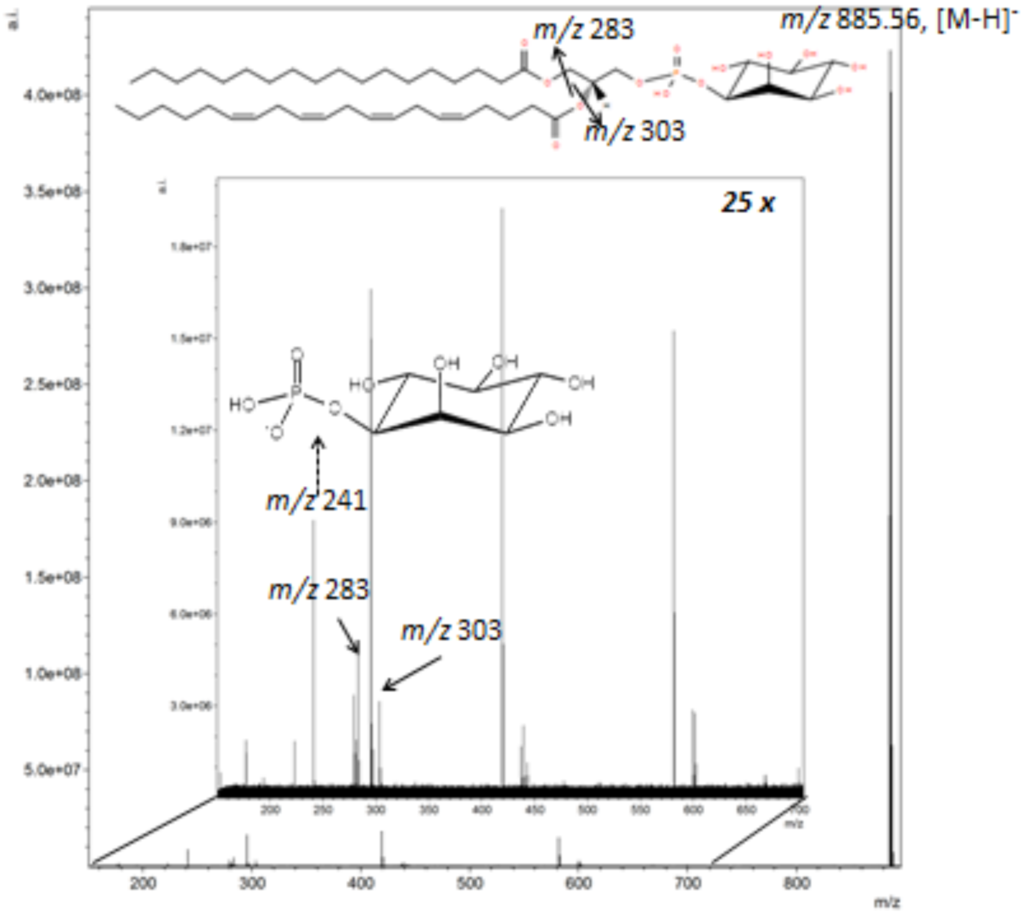

IV. Adjacent "Normal" Liver, Positive mode, *m/z* 764.7 $[M+Li]^+$, PC(16:0/18:2)

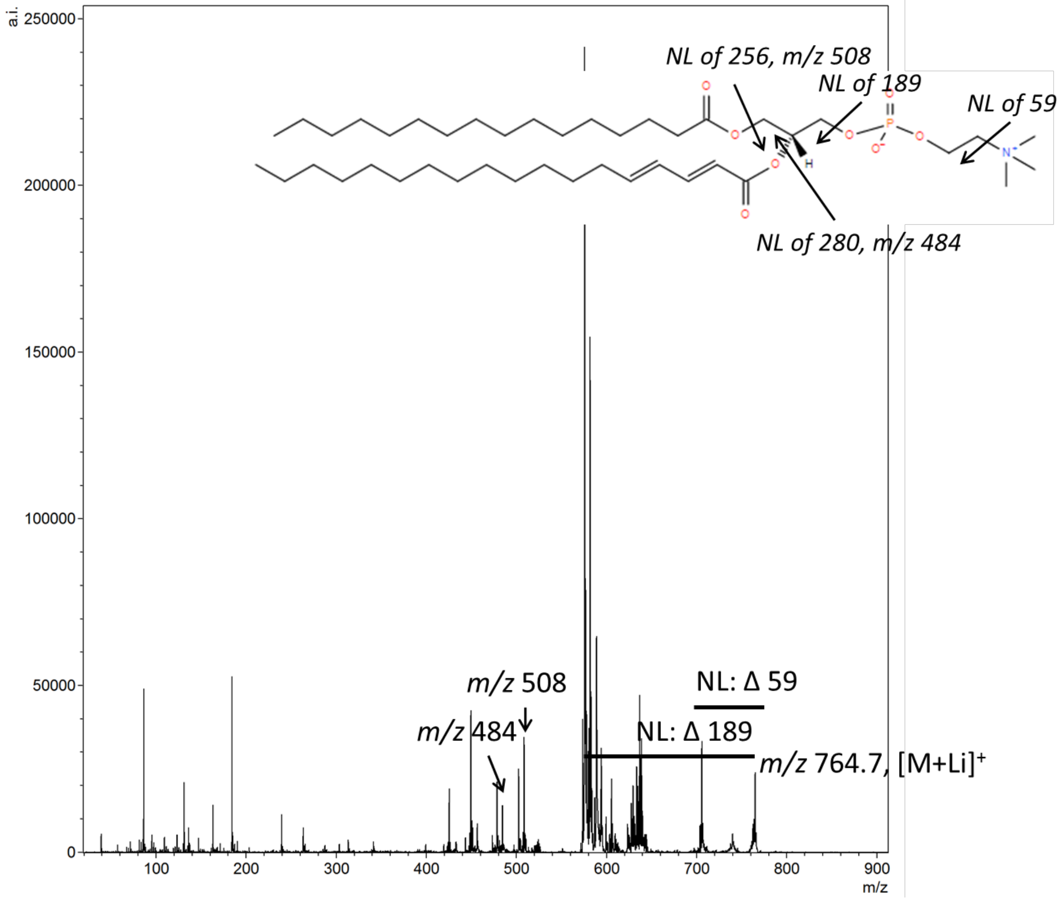

V. Viable Tumor, Negative mode, *m/z* 698.48 [M-H]$^-$, PE(p-16:0/18:2)

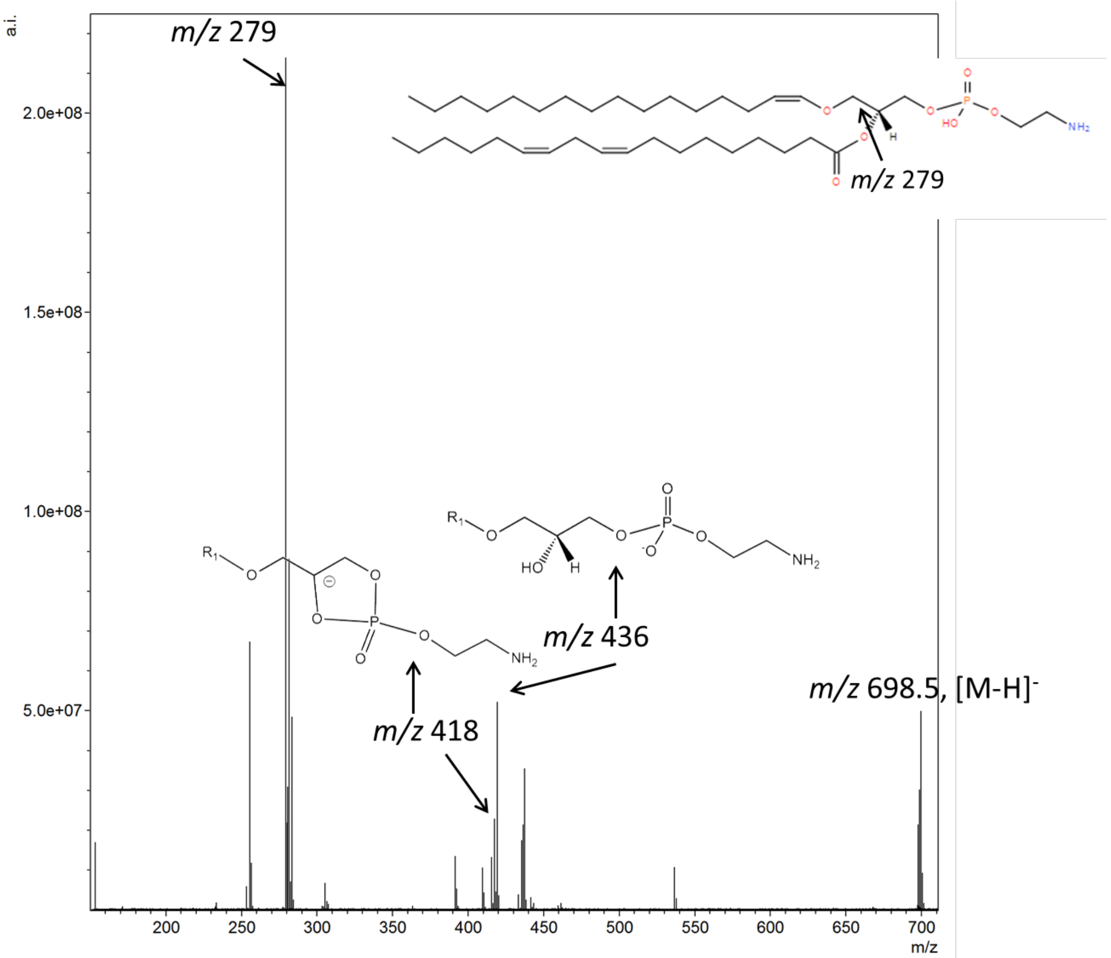

VI. Viable Tumor, Negative mode, *m/z* 700.51 [M-H]$^-$, PE(p-16:0/18:1)

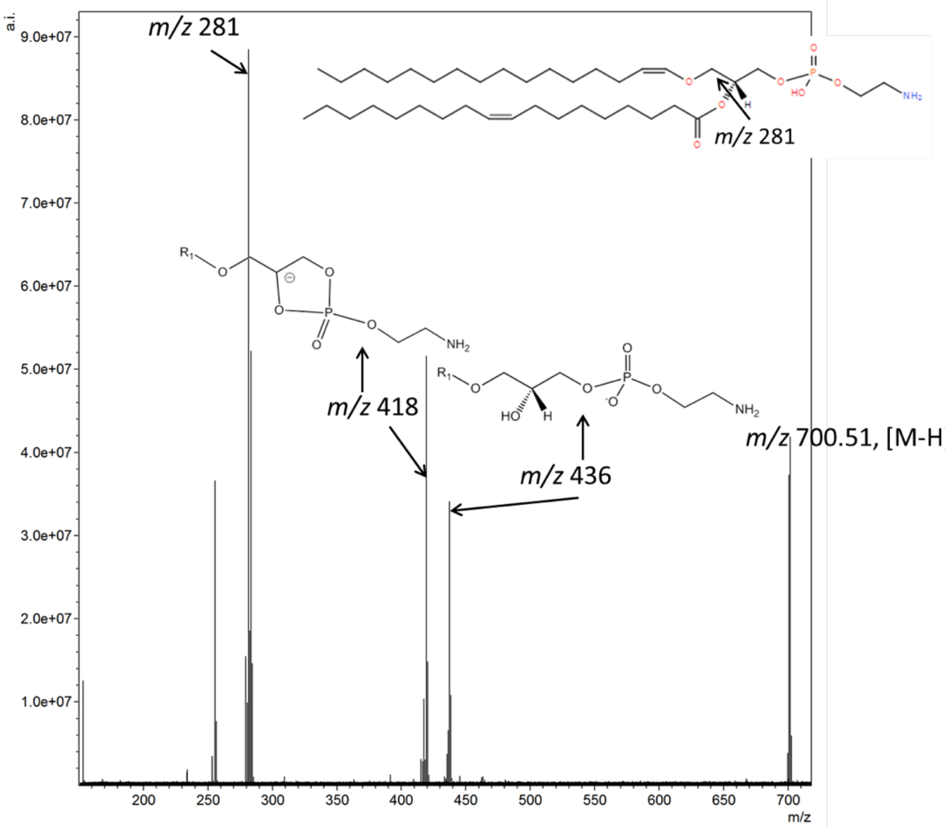

VII. Viable Tumor, Negative mode, *m/z* 835.53 [M-H]$^-$, PI(16:0/18:1)

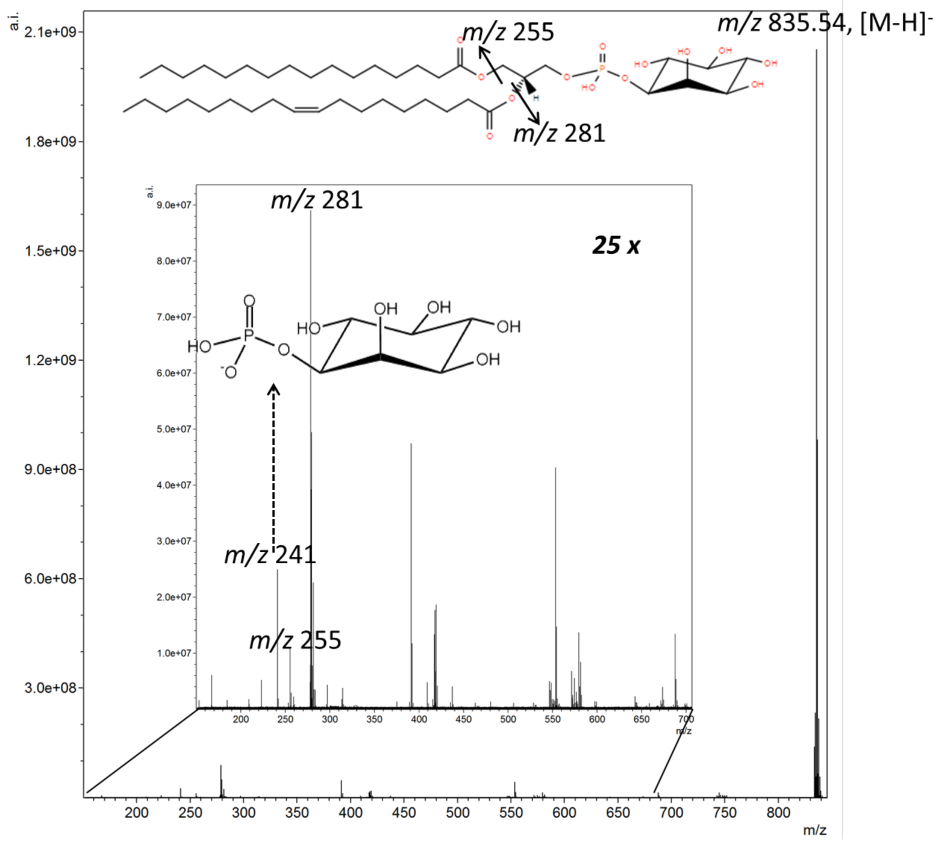

VIII. Viable Tumor, Positive mode, *m/z* 740.6 [M +Li]$^+$, PC(16:0/16:1)

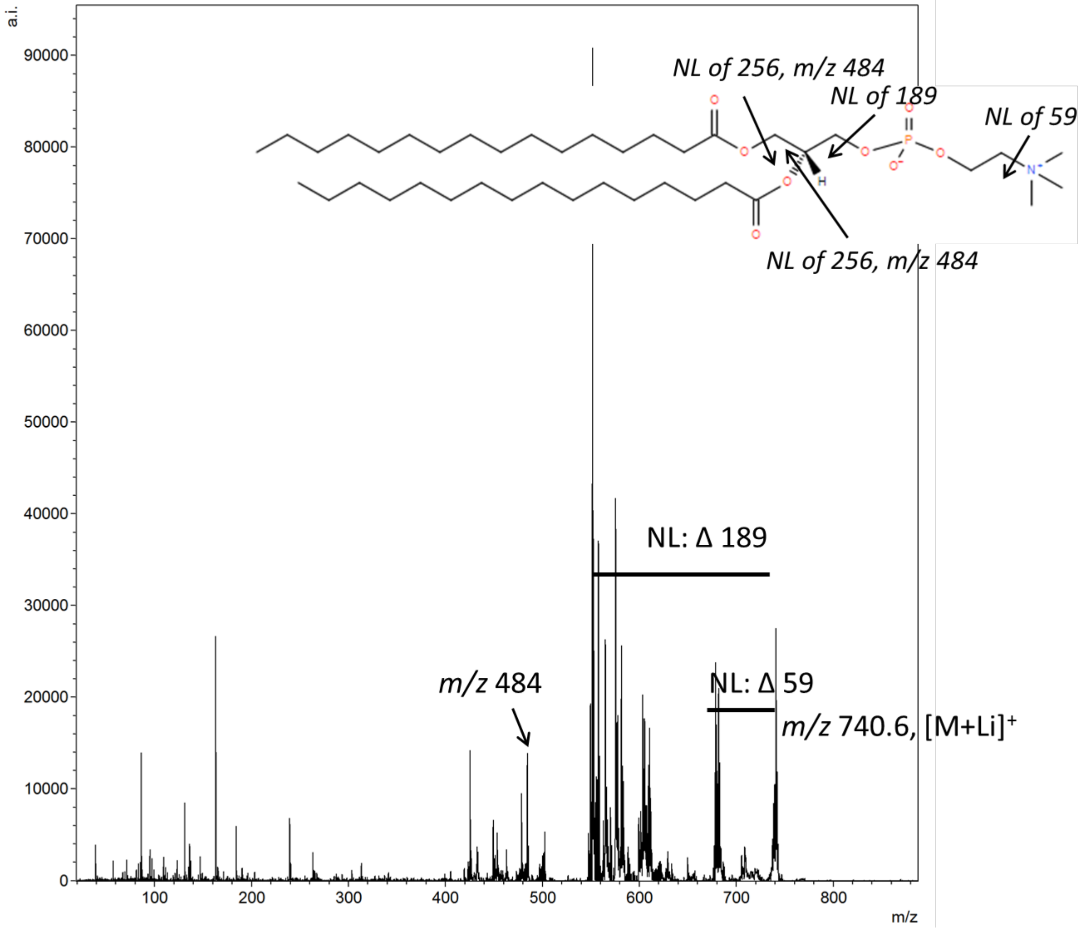

IX. **Inflammation, Negative Mode, *m/z* 722.49 [M-H]⁻, PE(p-16:0/ 20:4)**

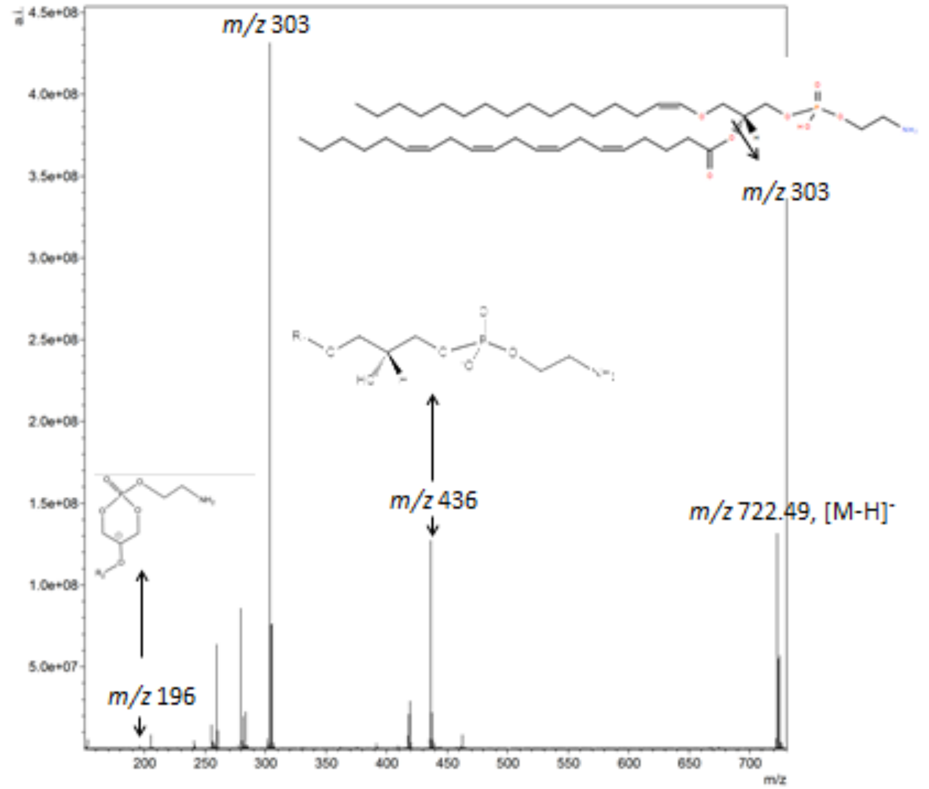

X. **Inflammation, Negative Mode, *m/z* 750.53 [M-H]⁻, PE(p-18:0/ 20:4)**

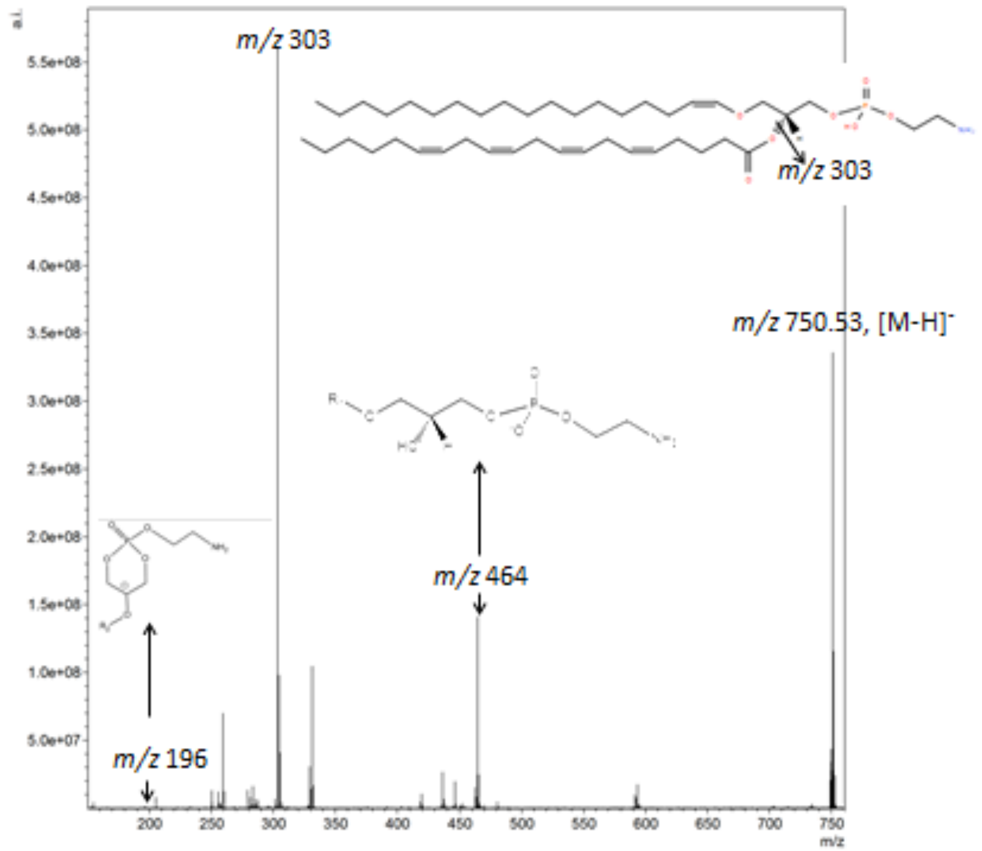

XI. **Inflammation, Positive Mode, *m/z* 738.6 [M +Li]$^{+}$, PC(16:0/ 16:0)**

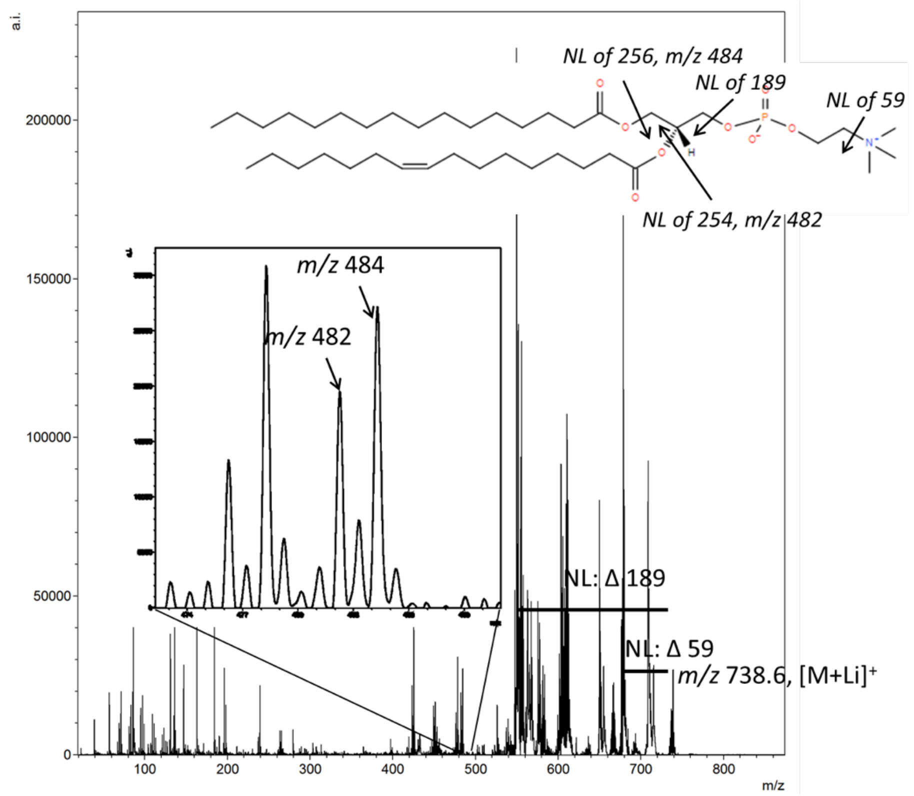

XII. **Necrosis, Positive Mode, *m/z* 709.6 [M +Li]$^{+}$, SM(d18:1/16:0)**

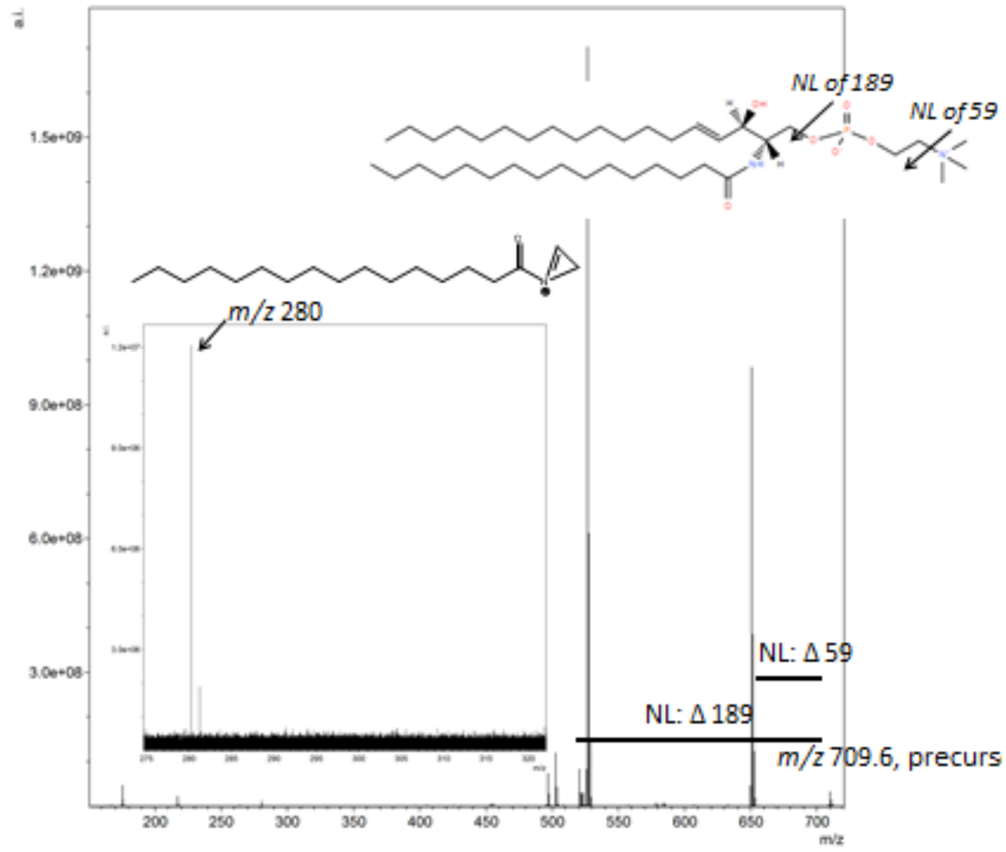

Supplemental Figure 6: MSI-H&E staining correlation for validation

Predictive MSI on testing set of CRC samples. (*a*) For all samples, a is the predicted MSI data at 100 µm. (*b*) large scale view of H&E staining of serial section. (*c-d*) zoom views of H&E for comparison.

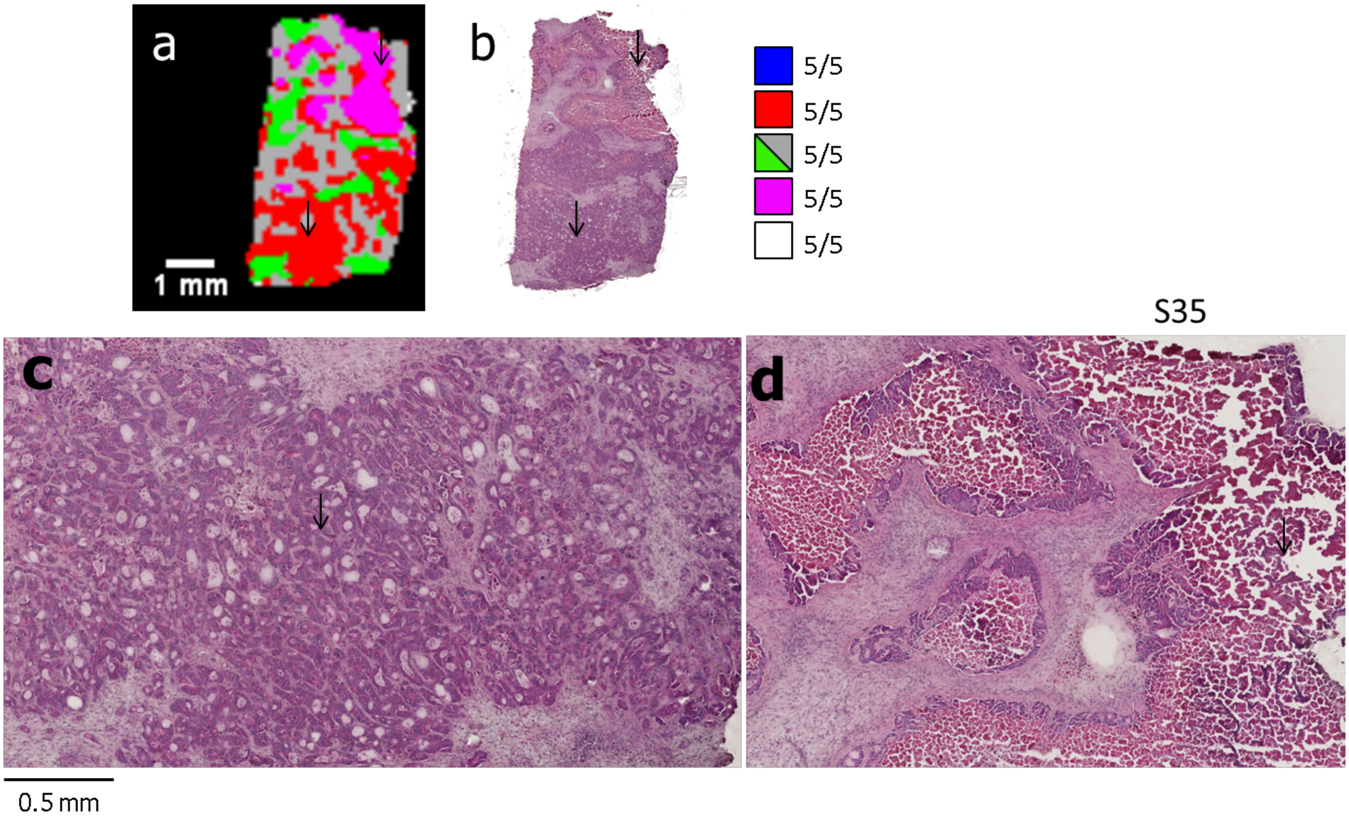

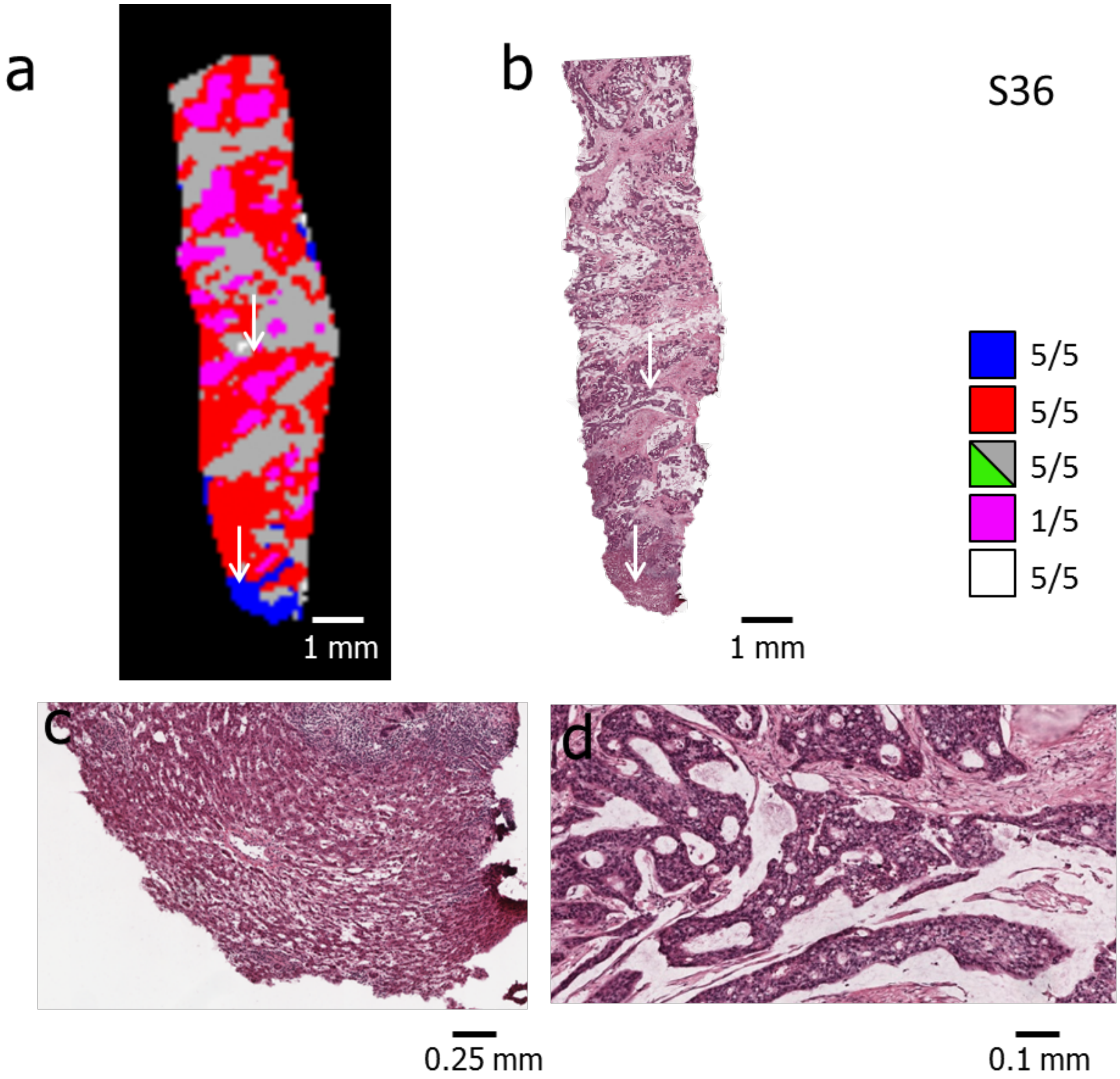

a
b
S36
1 mm
1 mm
5/5
5/5
5/5
1/5
5/5
c
d
0.25 mm
0.1 mm

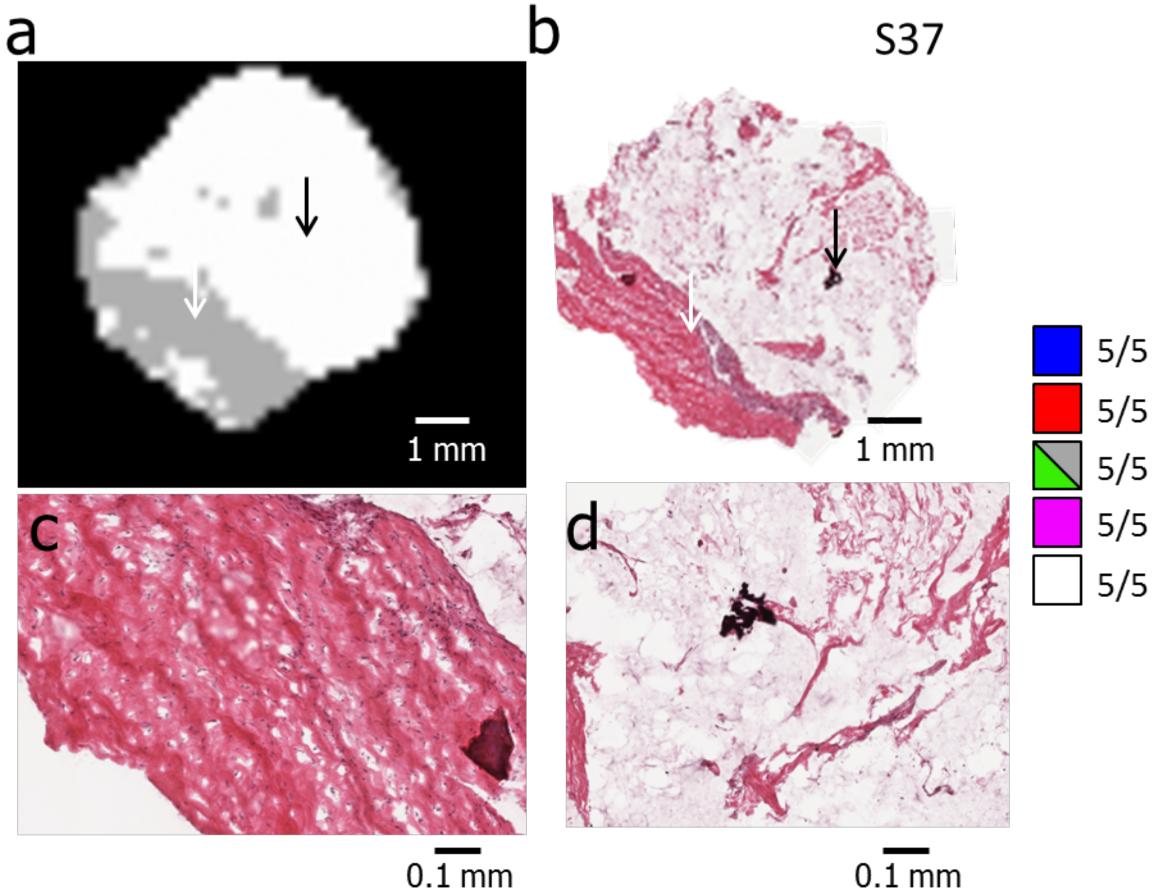
a
b
S37
1 mm
1 mm
c
d
0.1 mm
0.1 mm
5/5
5/5
5/5
5/5
5/5

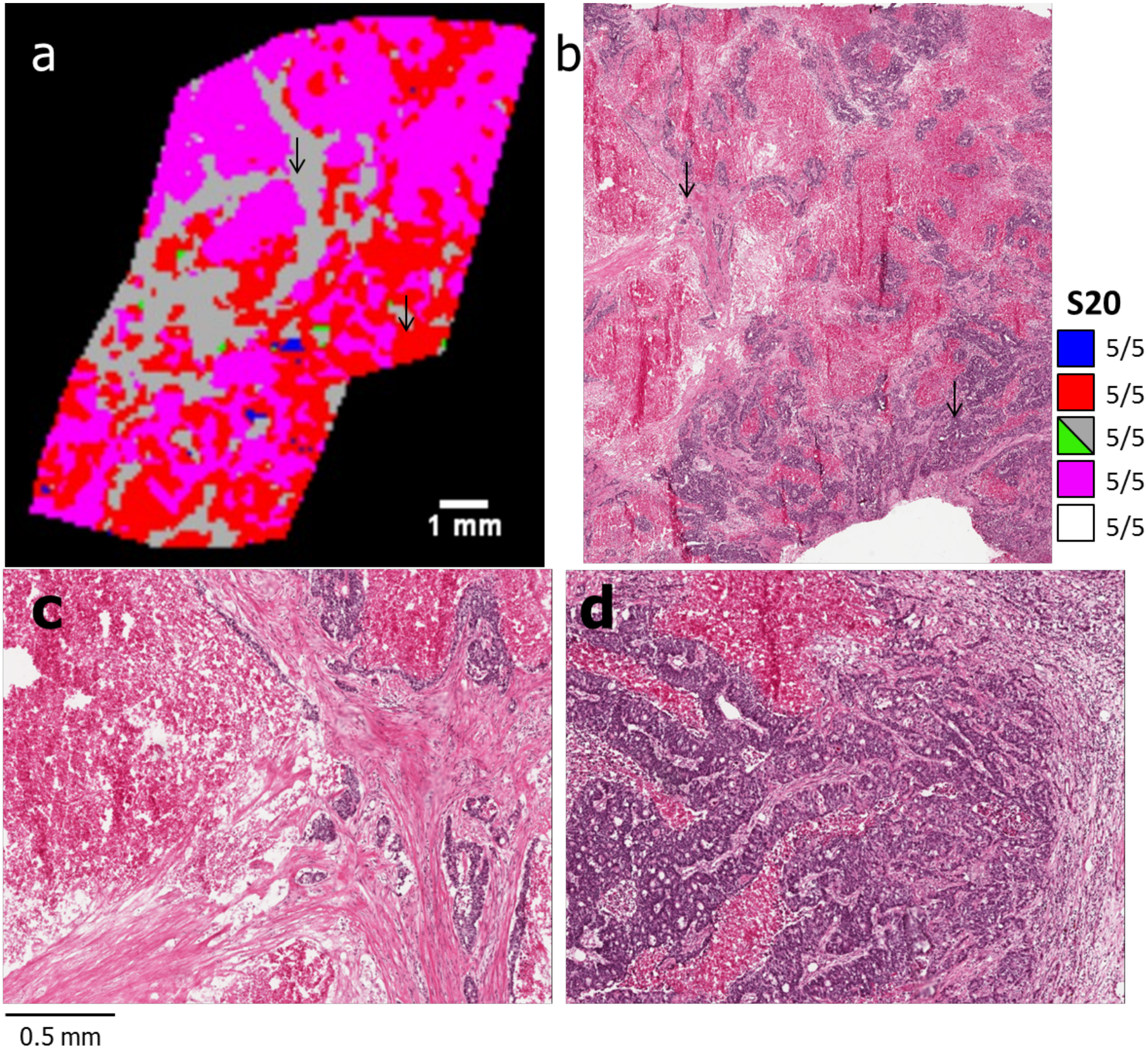
a
1 mm
b
S20
5/5
5/5
5/5
5/5
5/5
c
d
0.5 mm

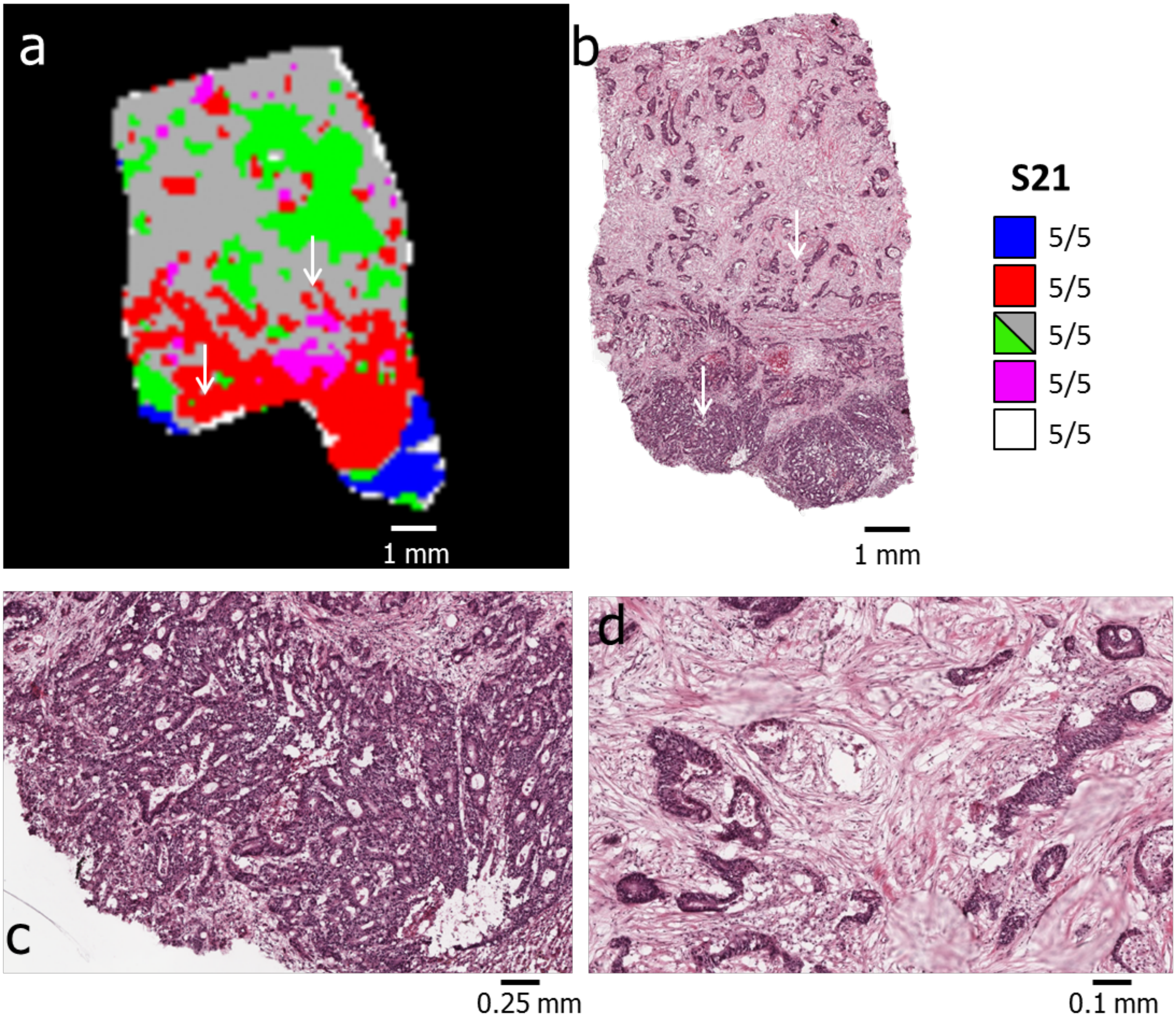
a
1 mm
b
1 mm
S21
5/5
5/5
5/5
5/5
5/5
c
0.25 mm
d
0.1 mm

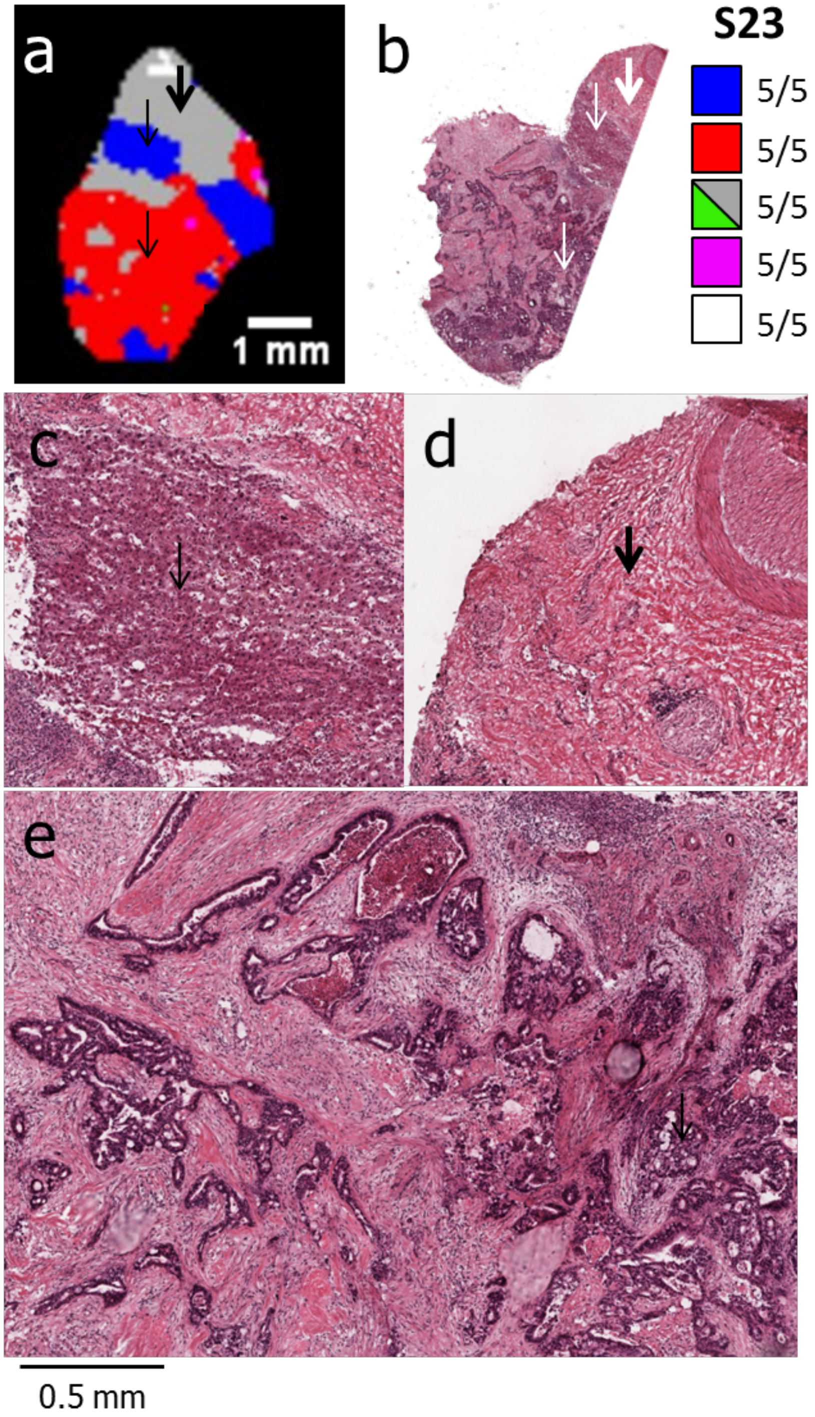

a
1 mm
b
S23
5/5
5/5
5/5
5/5
5/5
c
d
e
0.5 mm

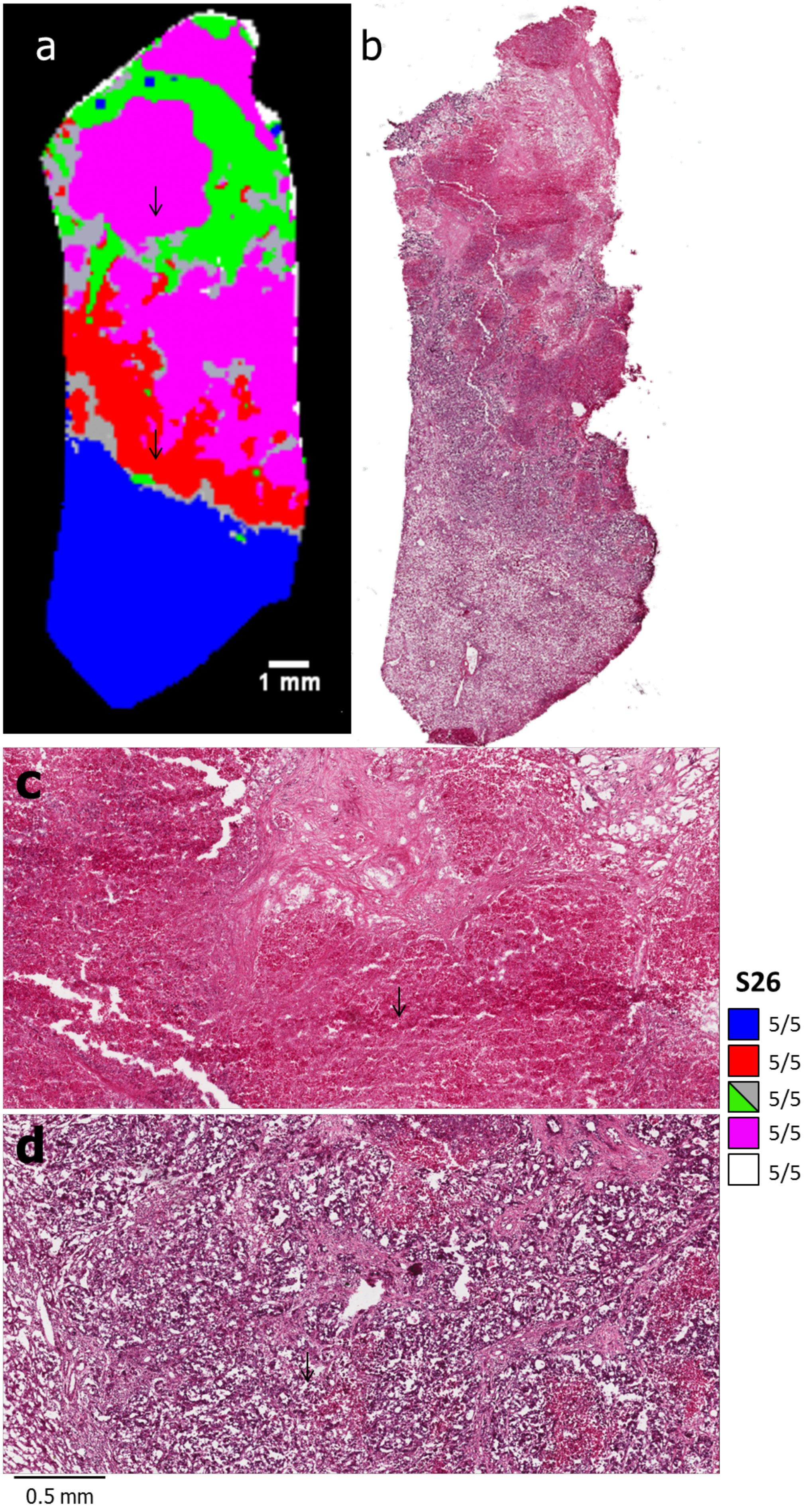
a
b
1 mm
c
d
0.5 mm
S26
5/5
5/5
5/5
5/5
5/5

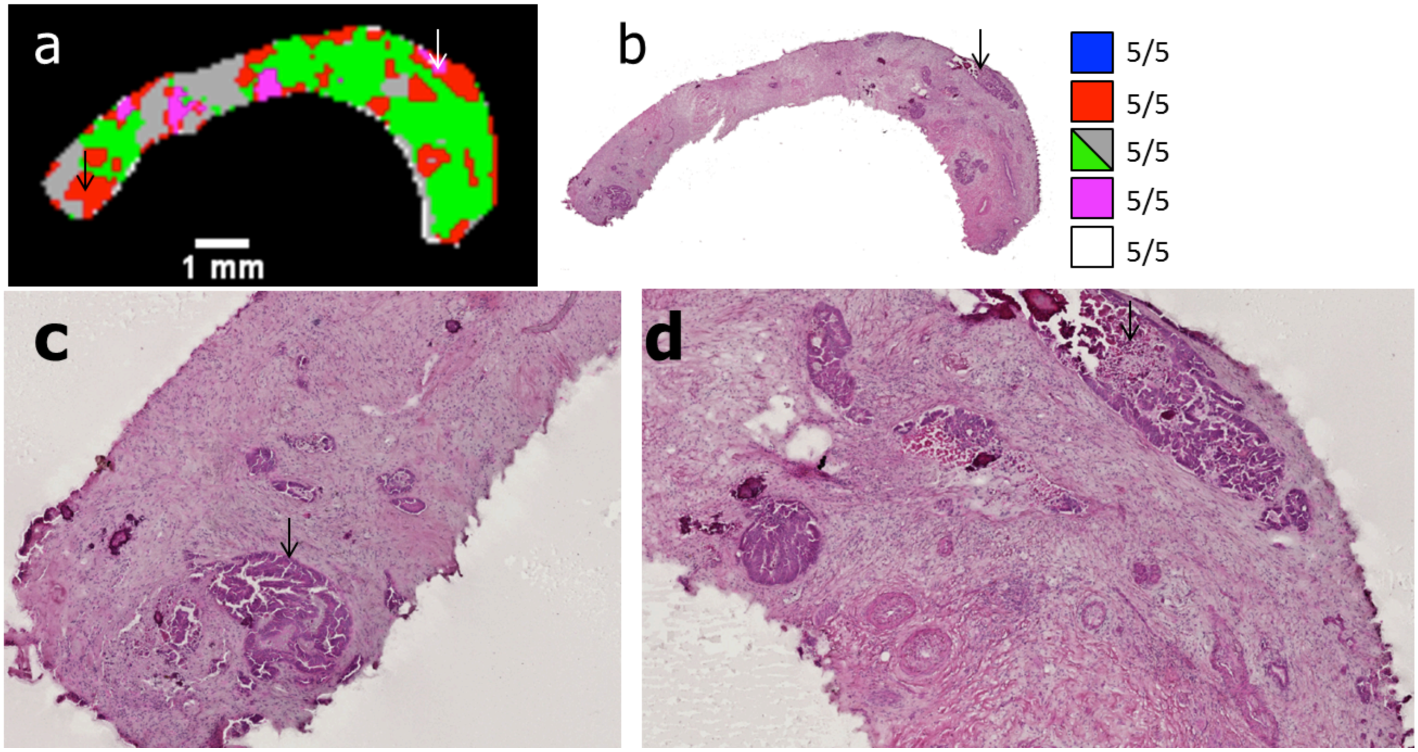
a
1 mm
b
5/5
5/5
5/5
5/5
5/5
c
d

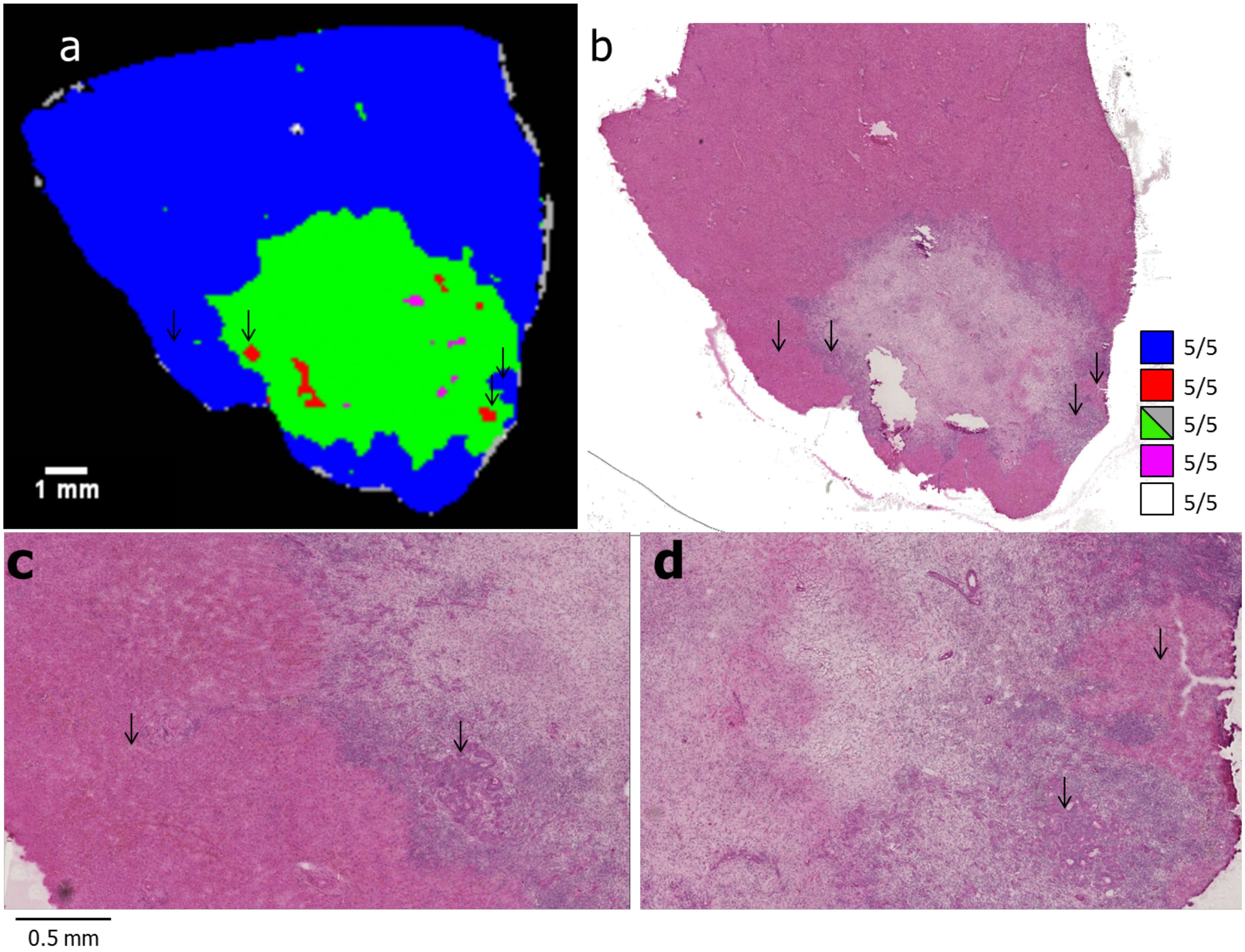
a
1 mm
b
5/5
5/5
5/5
5/5
5/5
c
d
0.5 mm

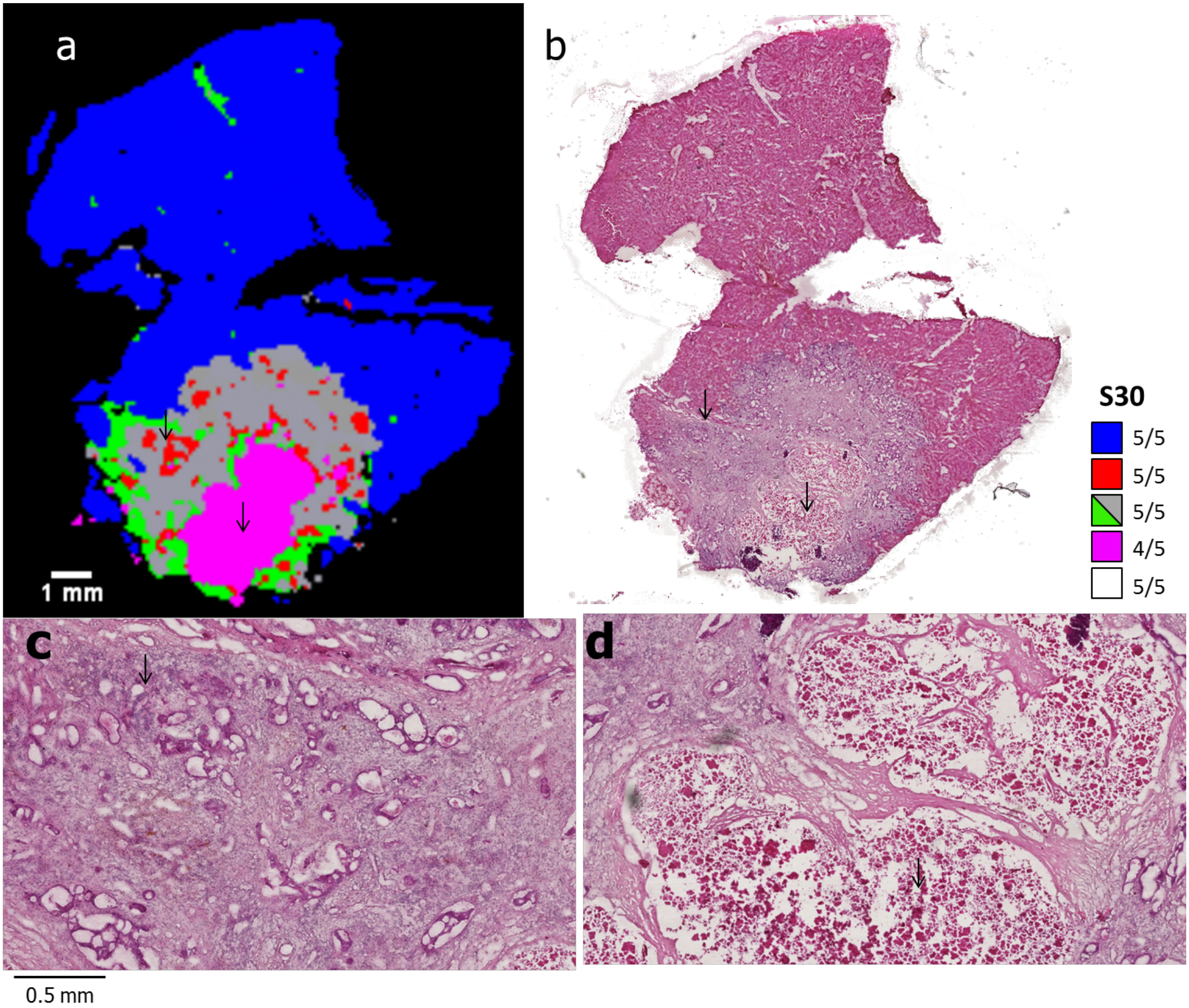
a
1 mm
b
S30
5/5
5/5
5/5
4/5
5/5
c
d
0.5 mm

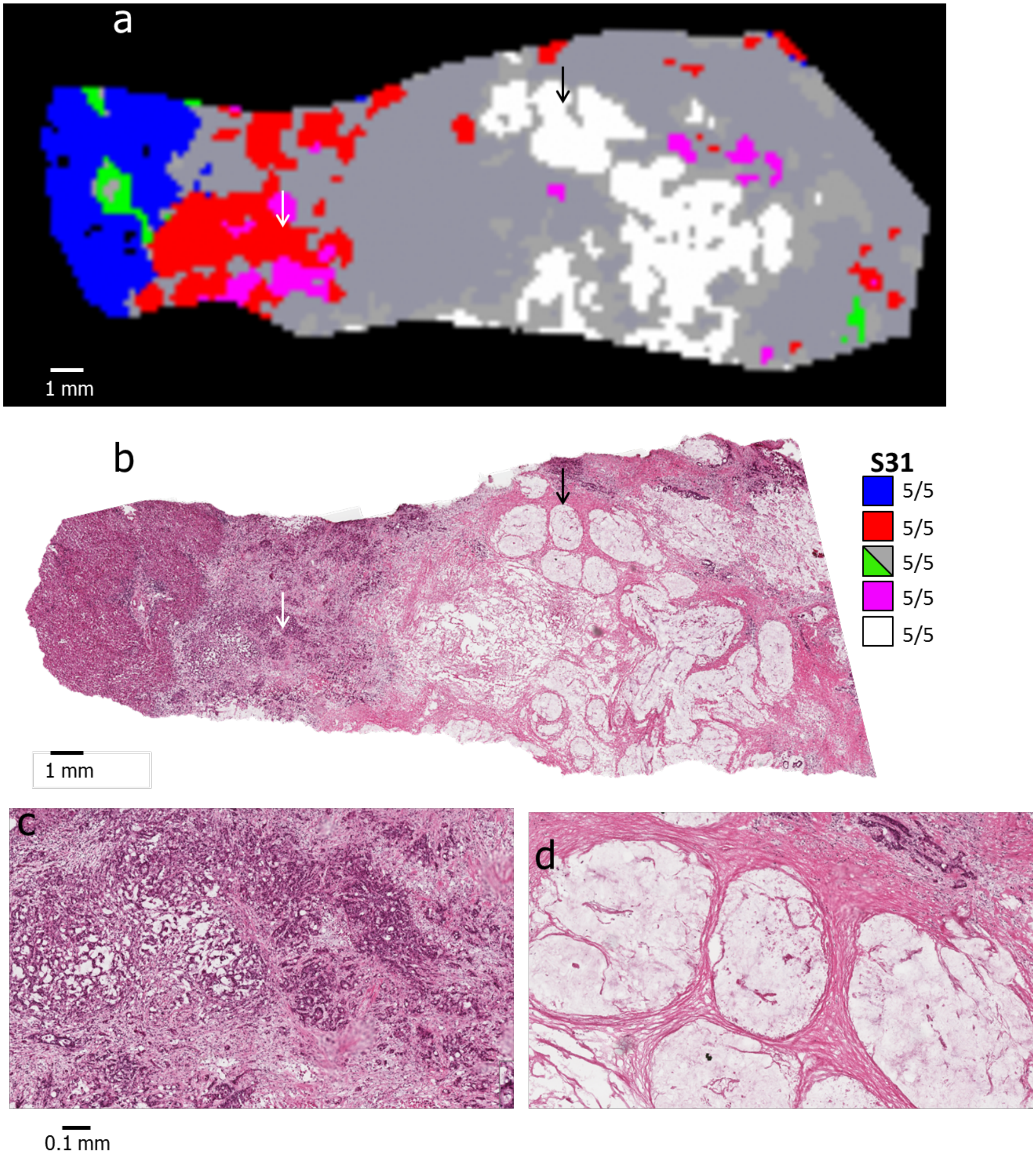
a
1 mm
b
S31
5/5
5/5
5/5
5/5
5/5
1 mm
c
d
0.1 mm

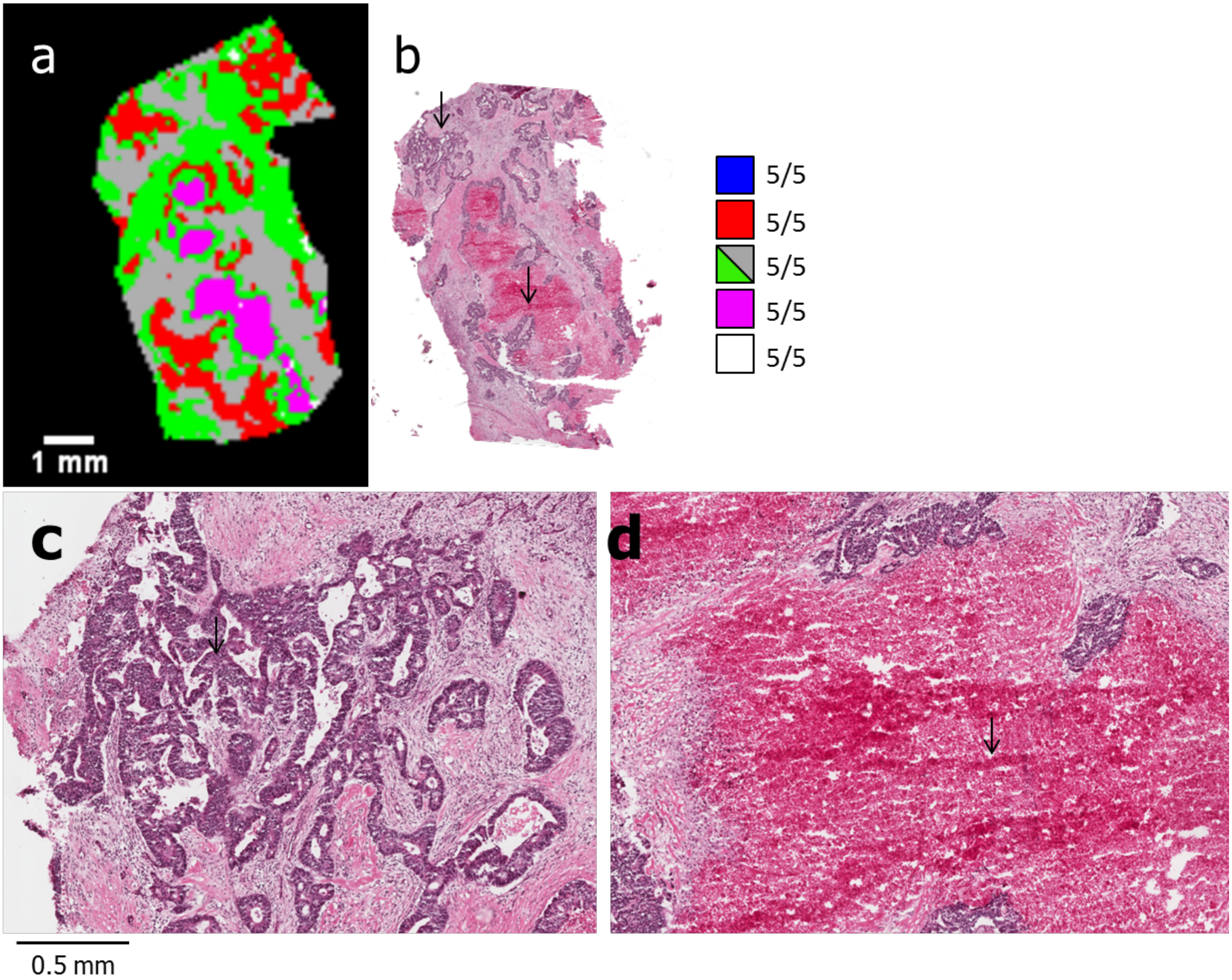
a
1 mm
b
5/5
5/5
5/5
5/5
5/5
c
d
0.5 mm

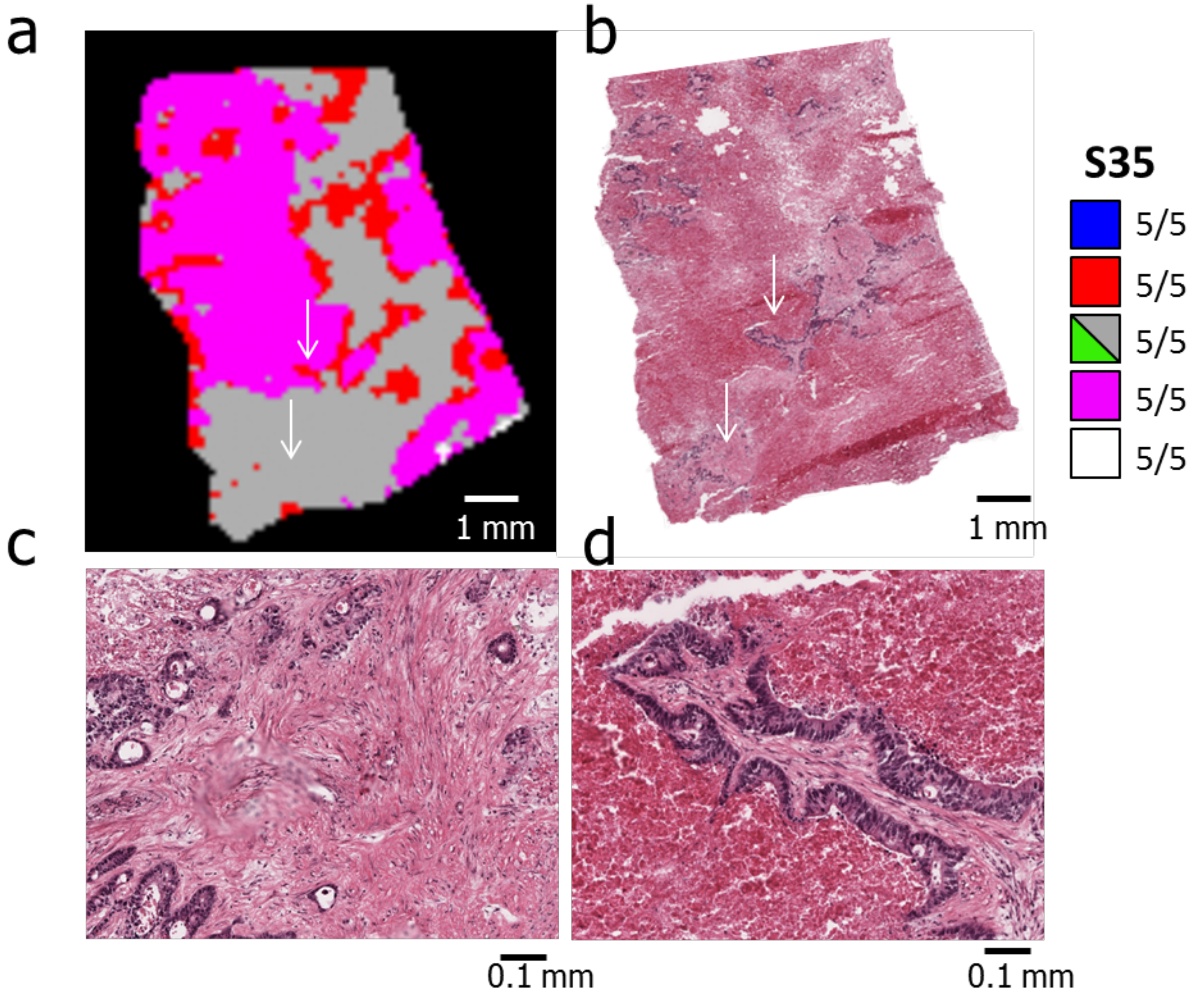
a
b
c
d
S35
5/5
5/5
5/5
5/5
5/5
1 mm
1 mm
0.1 mm
0.1 mm

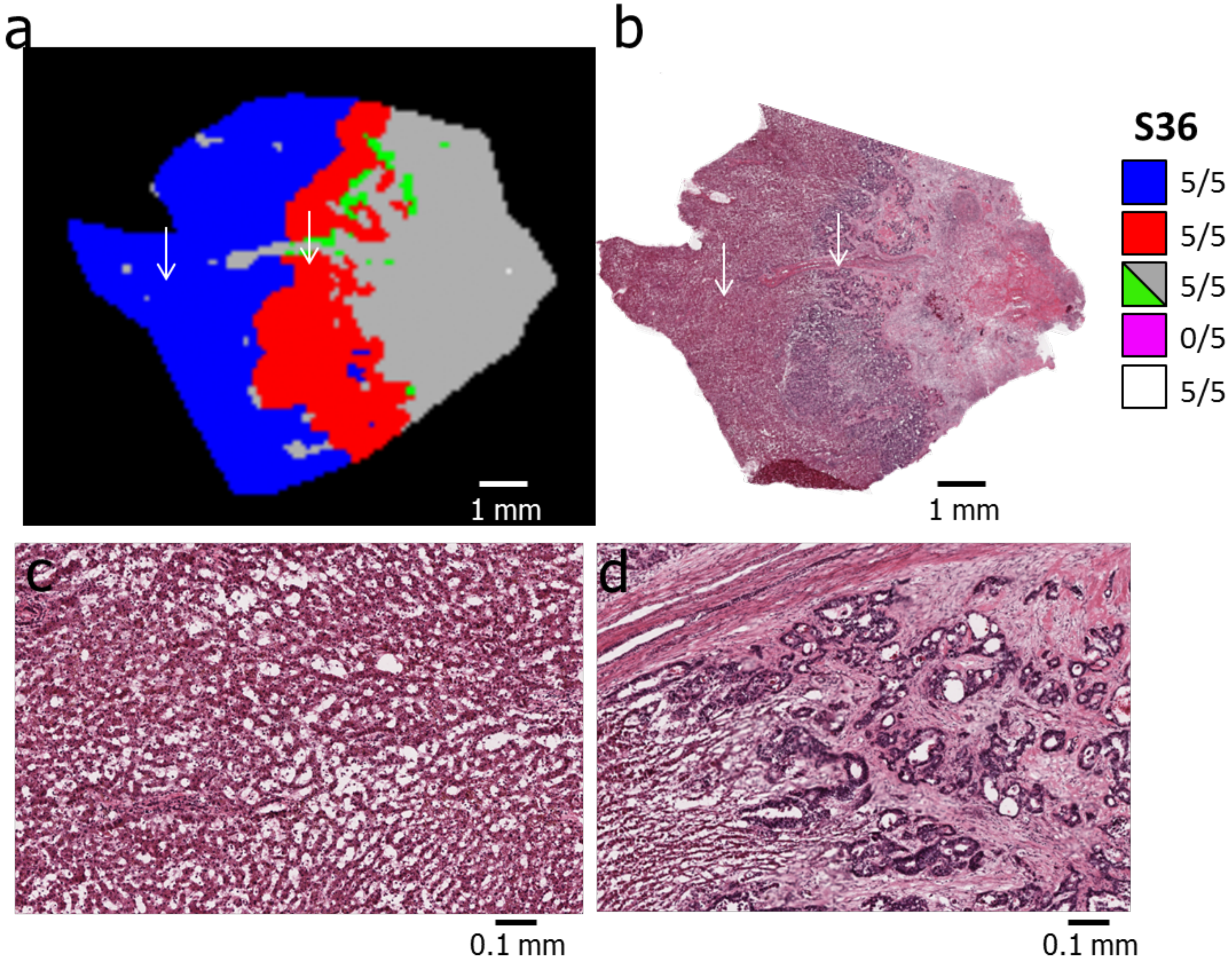

a
b
S36
5/5
5/5
5/5
0/5
5/5
1 mm
1 mm
c
d
0.1 mm
0.1 mm

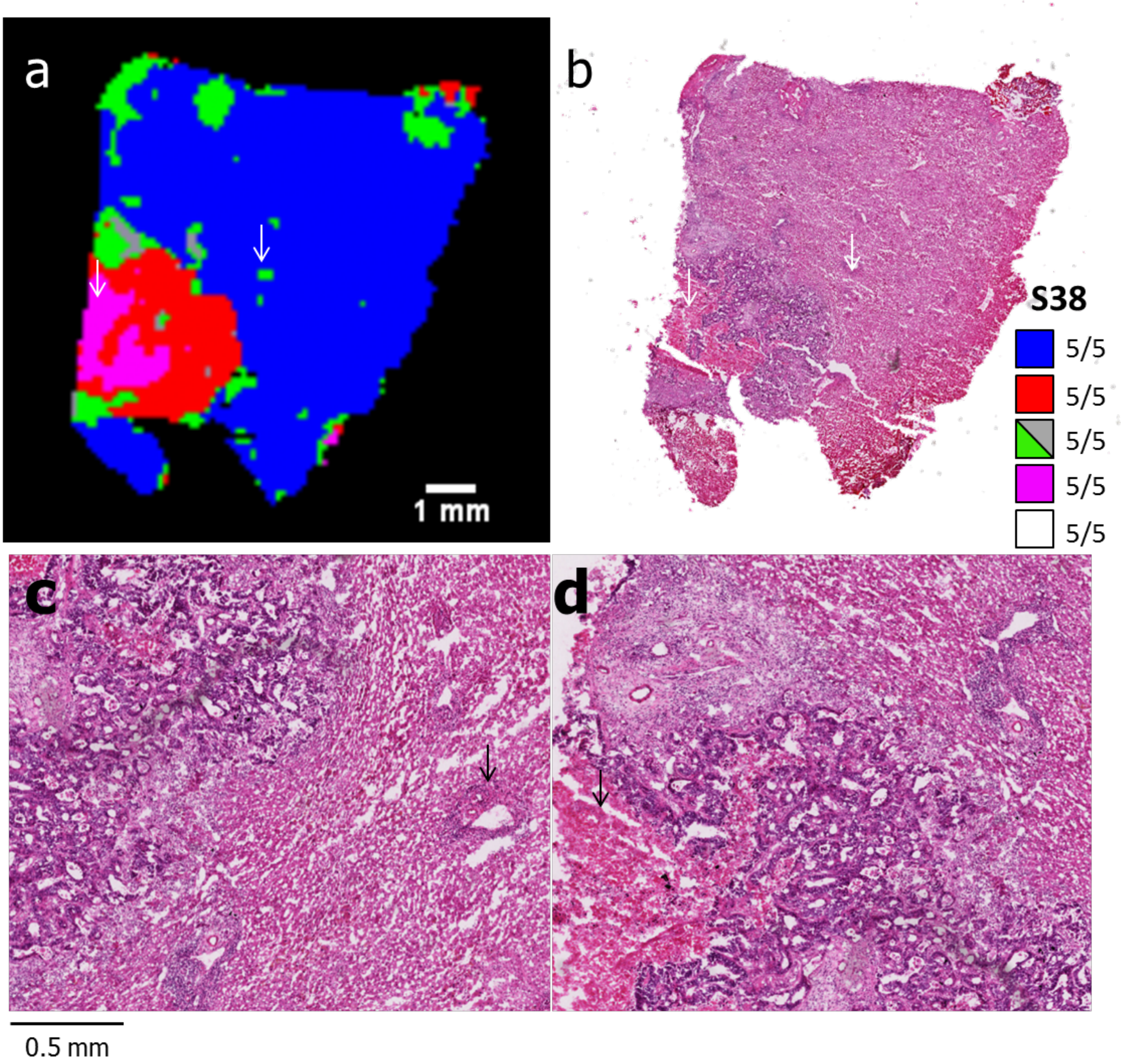
a
1 mm
b
S38
5/5
5/5
5/5
5/5
5/5
c
d
0.5 mm

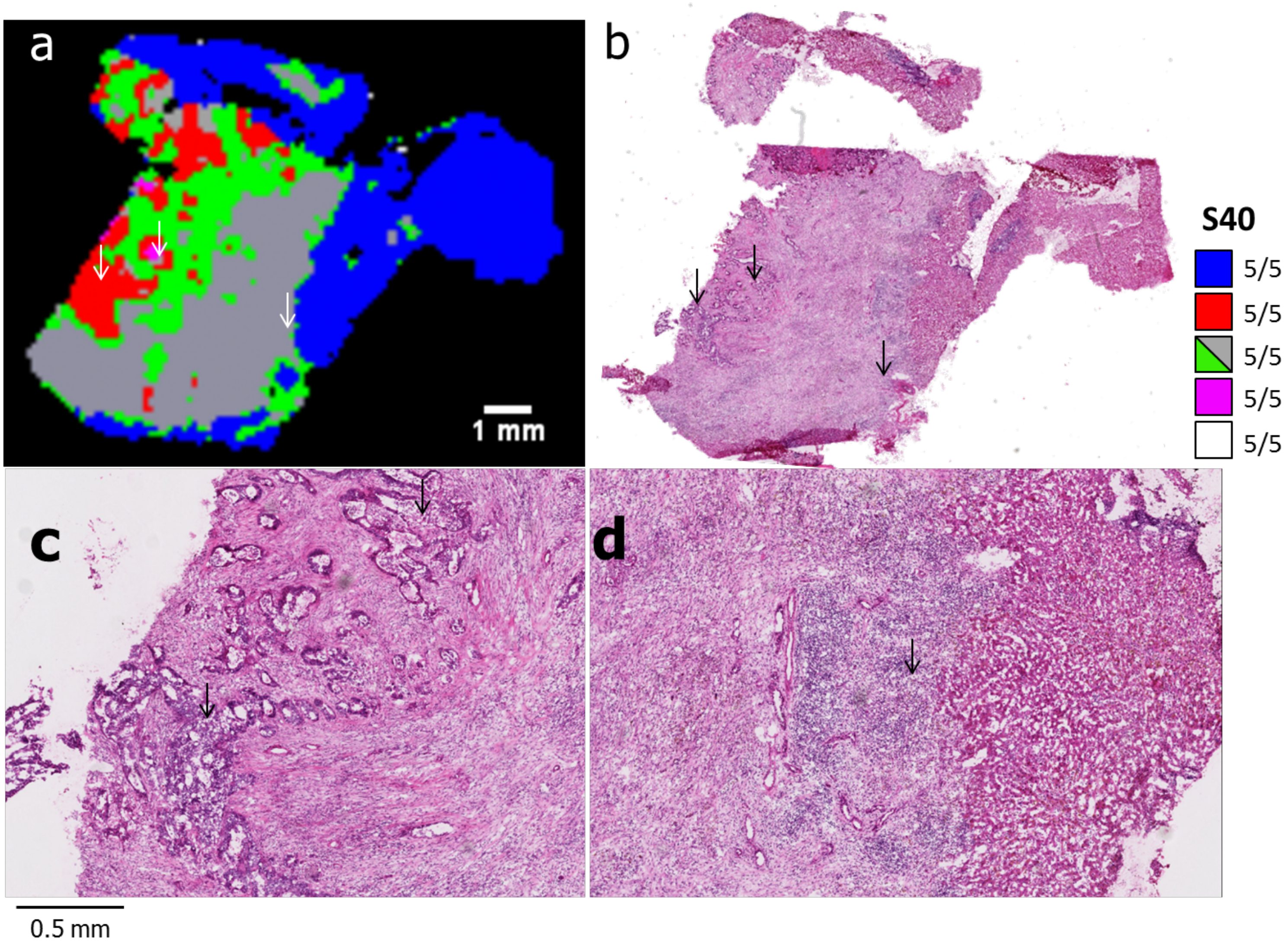

a
b
c
d
1 mm
S40
5/5
5/5
5/5
5/5
5/5
0.5 mm

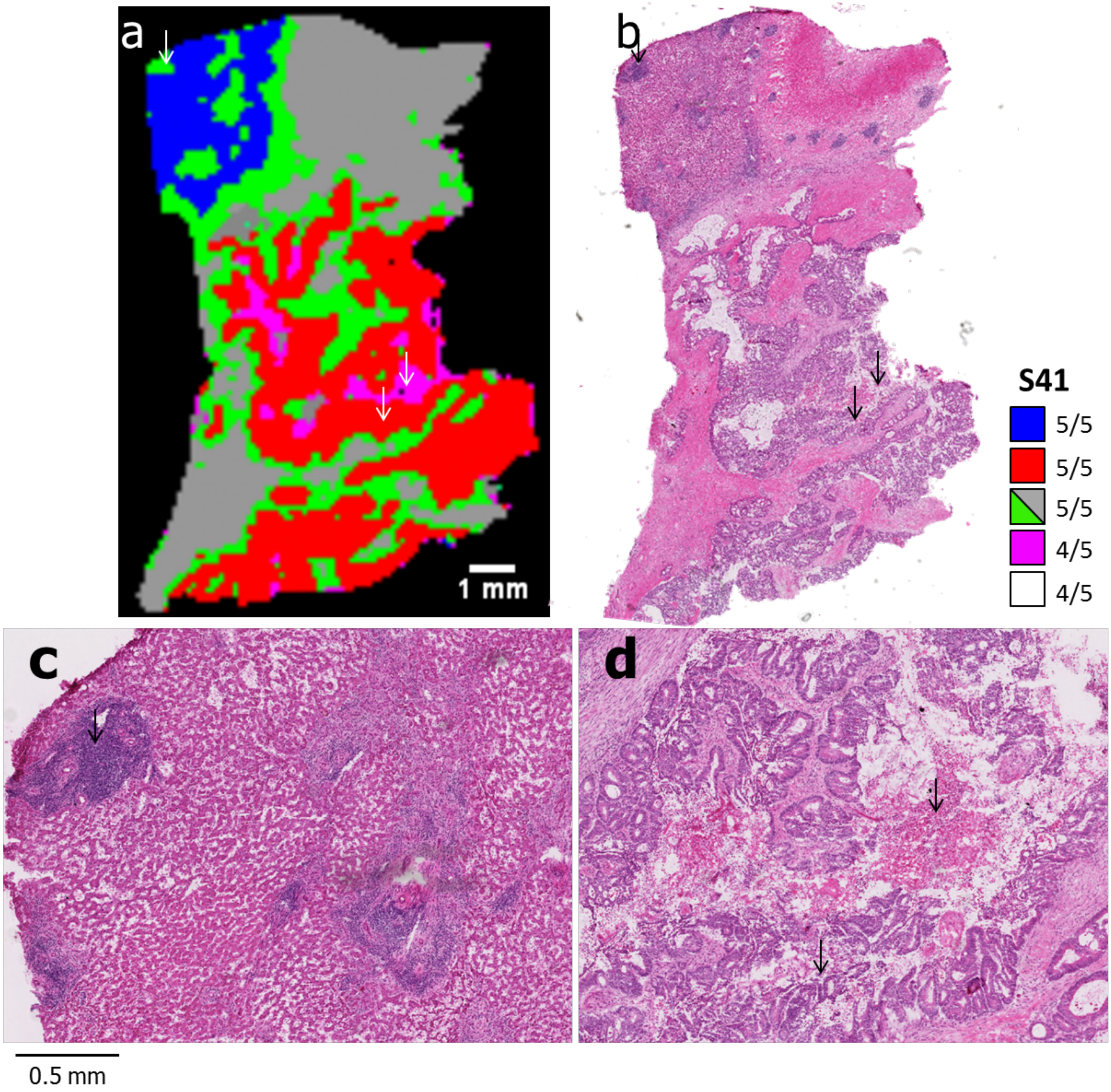
a
1 mm
b
S41
5/5
5/5
5/5
4/5
4/5
c
d
0.5 mm

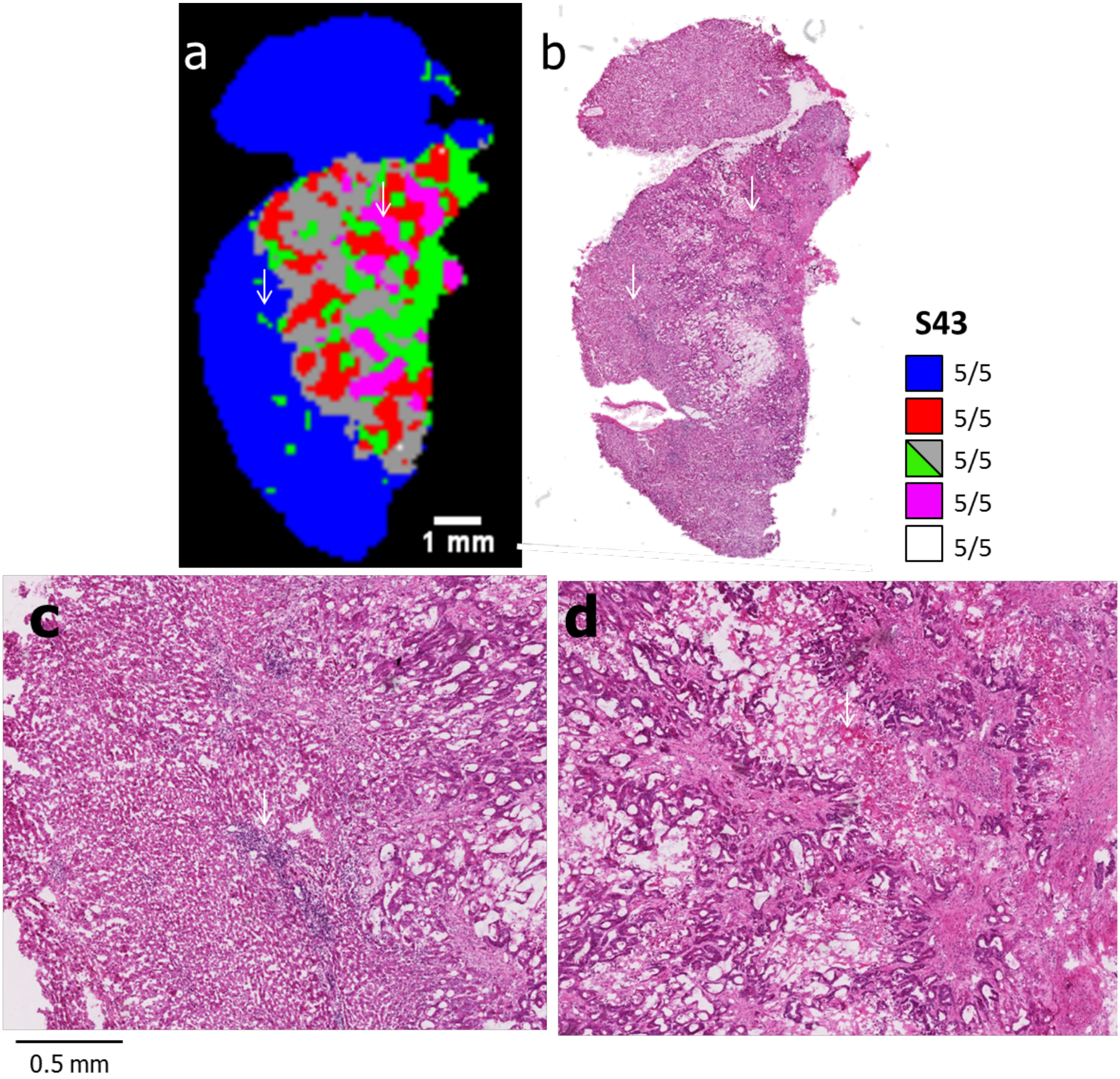
a
1 mm
b
S43
5/5
5/5
5/5
5/5
5/5
c
d
0.5 mm

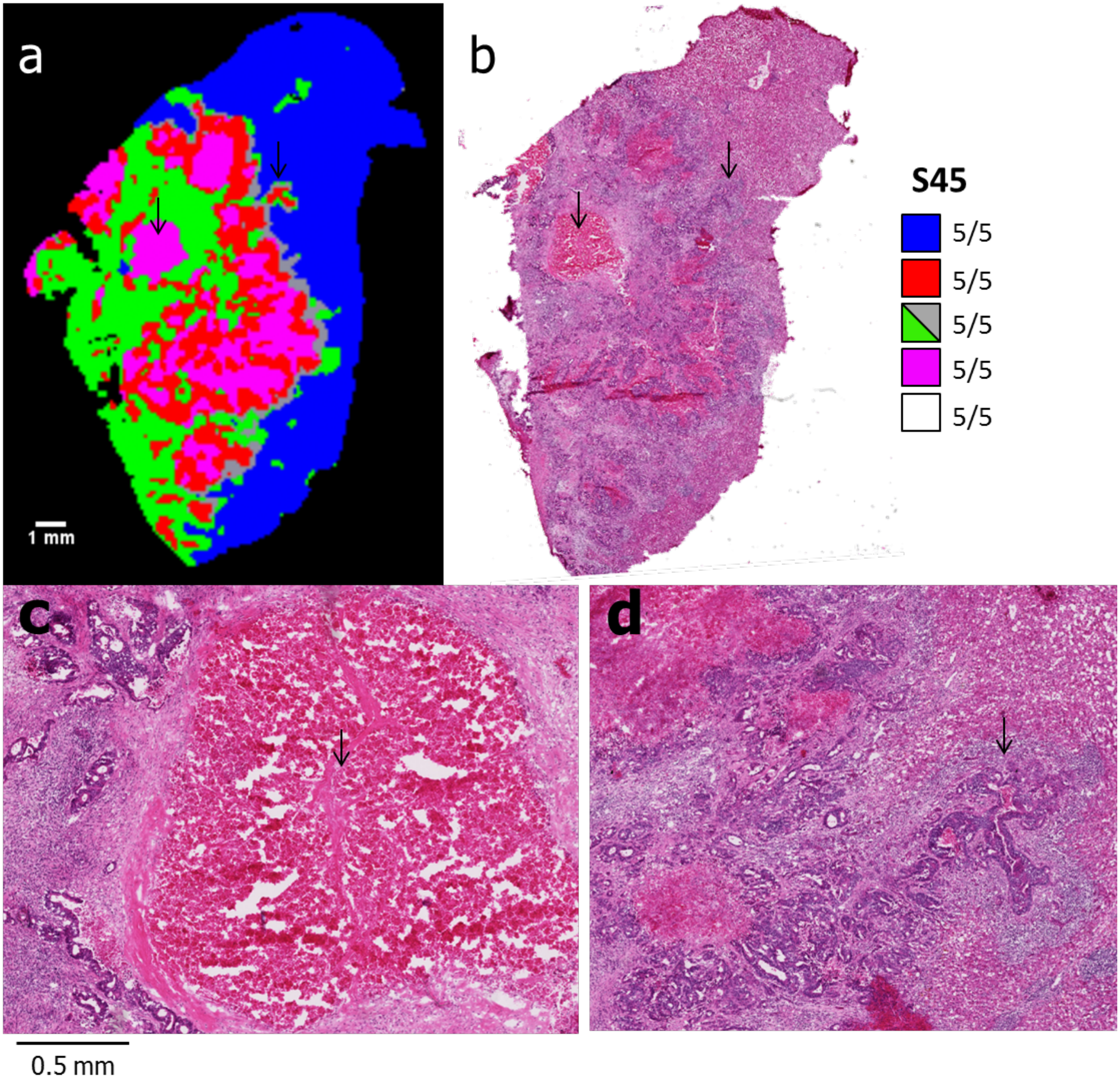
a
1 mm
b
S45
5/5
5/5
5/5
5/5
5/5
c
d
0.5 mm

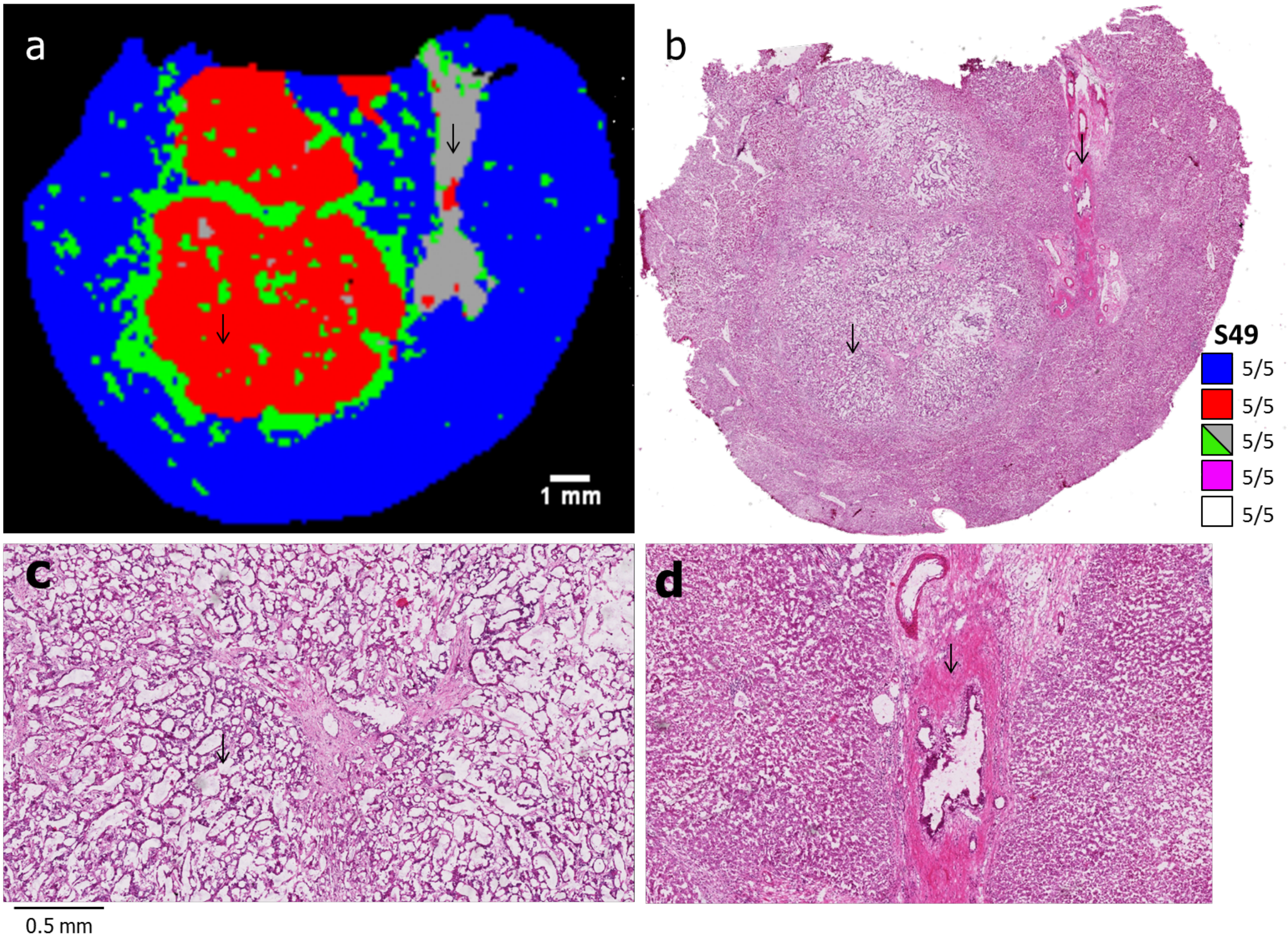
a
1 mm
b
S49
5/5
5/5
5/5
5/5
5/5
c
d
0.5 mm

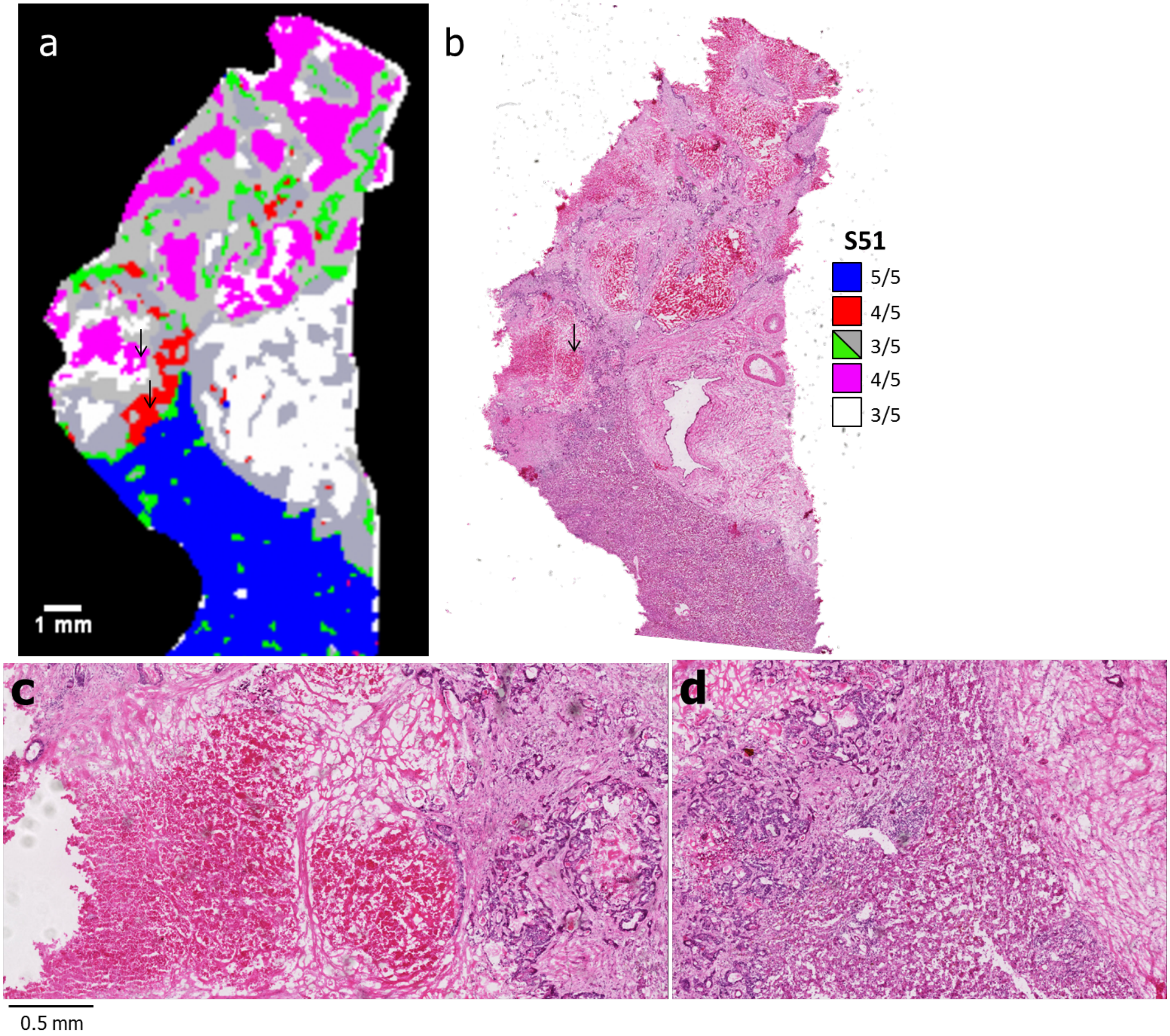

a
b
S51
5/5
4/5
3/5
4/5
3/5
1 mm
c
d
0.5 mm

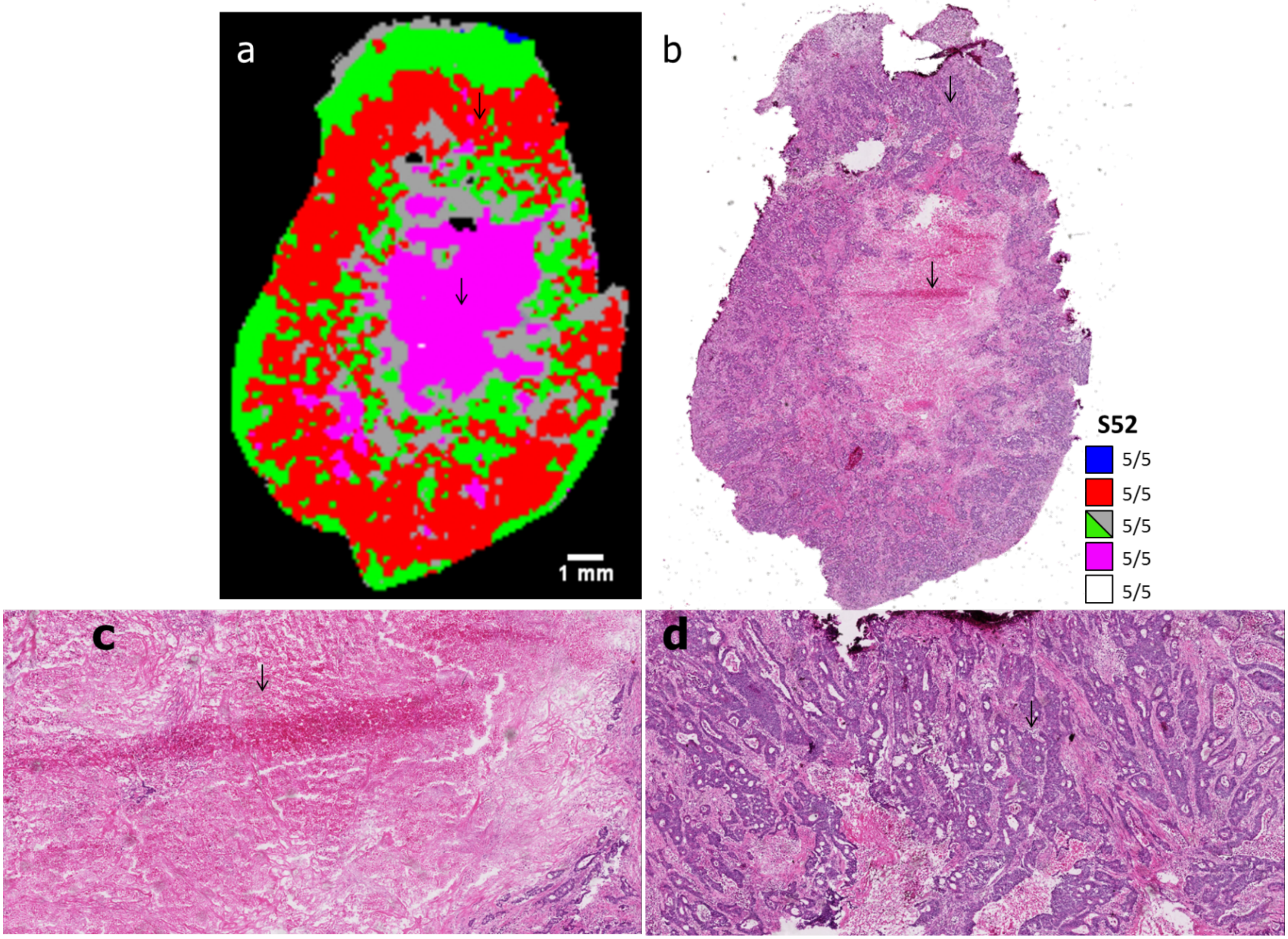

a
1 mm
b
S52
5/5
5/5
5/5
5/5
5/5
c
d

Supplemental Figure 7:

Profiling and lithiated MS/MS of ions co-localized in areas of CRCLM usual necrosis.

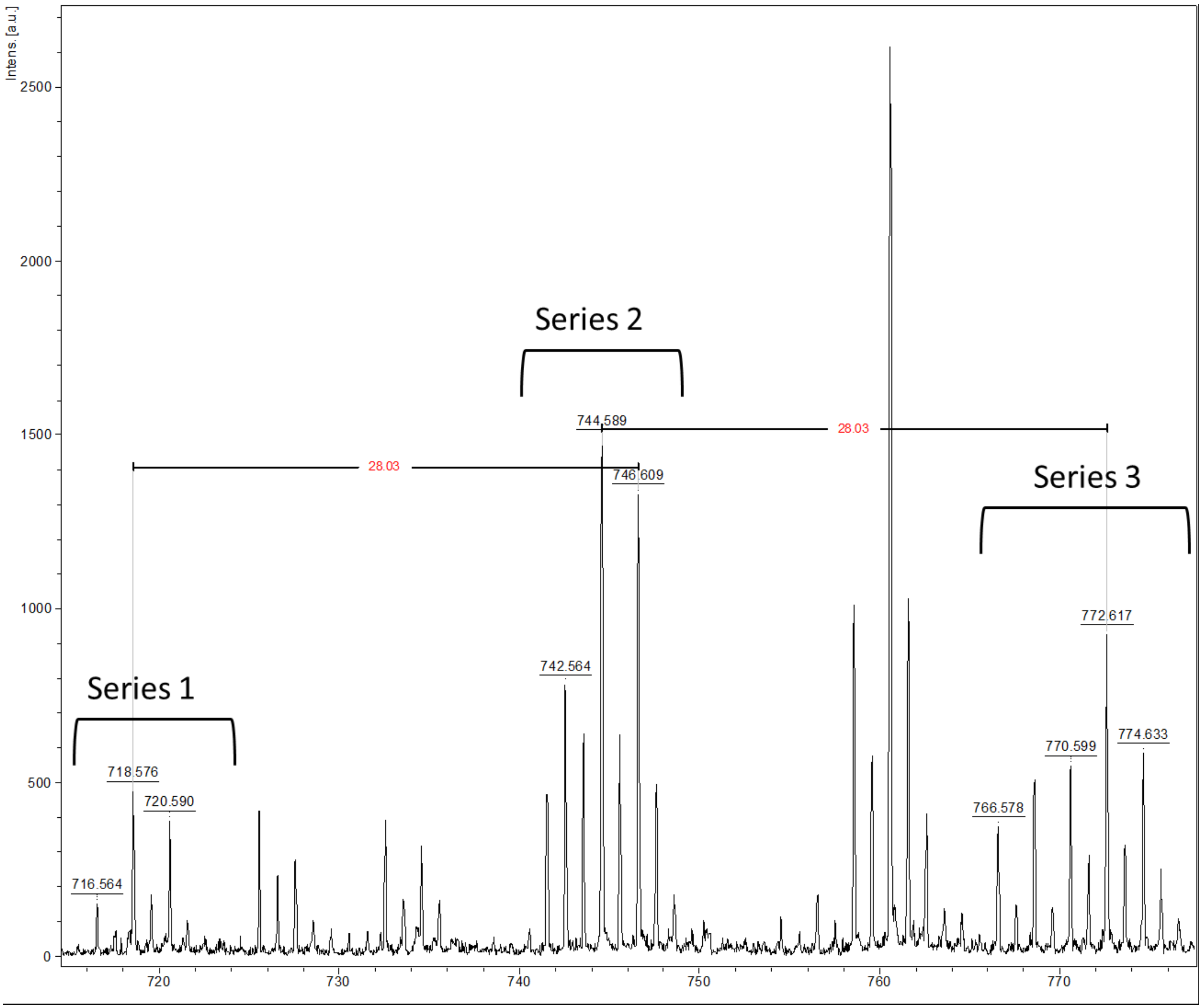

Profile of the three plasmalogen series. Annotations added for 28 Da FA chain elongations between series

.

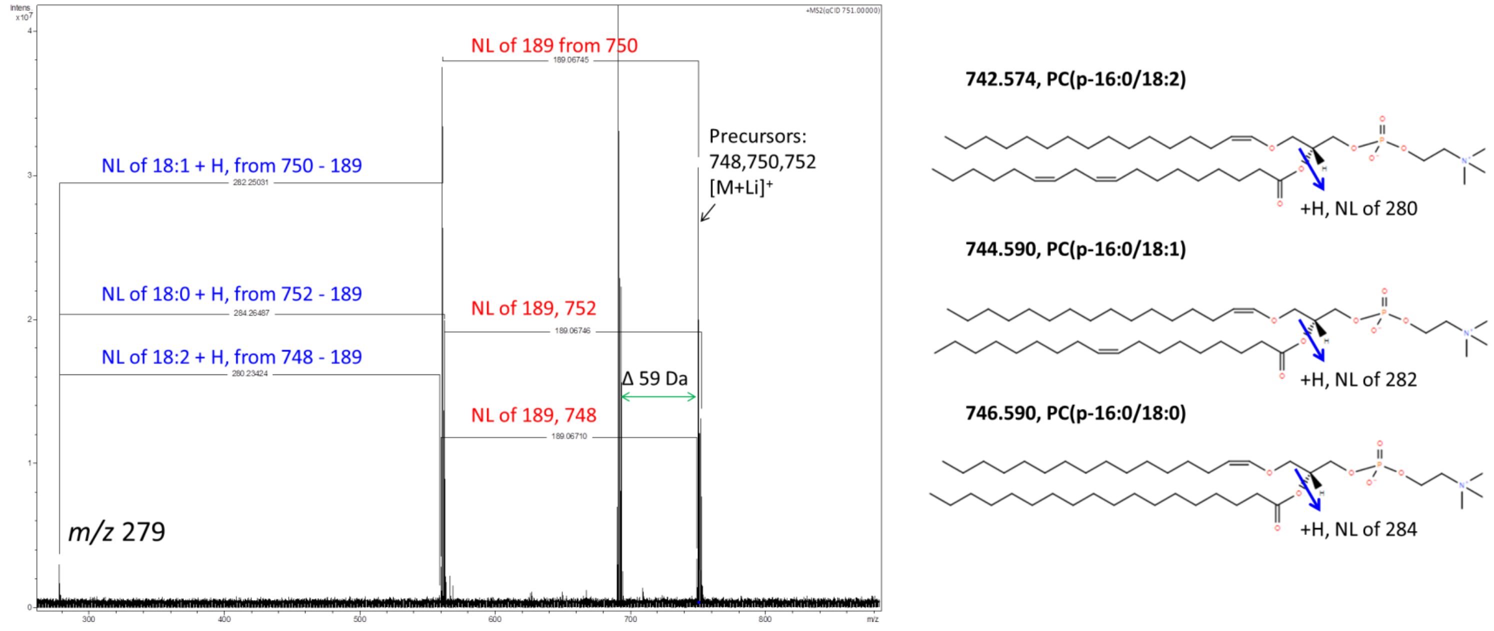

MS/MS of Series 2, isolation window 747.5-753.5. Three precursor ions.

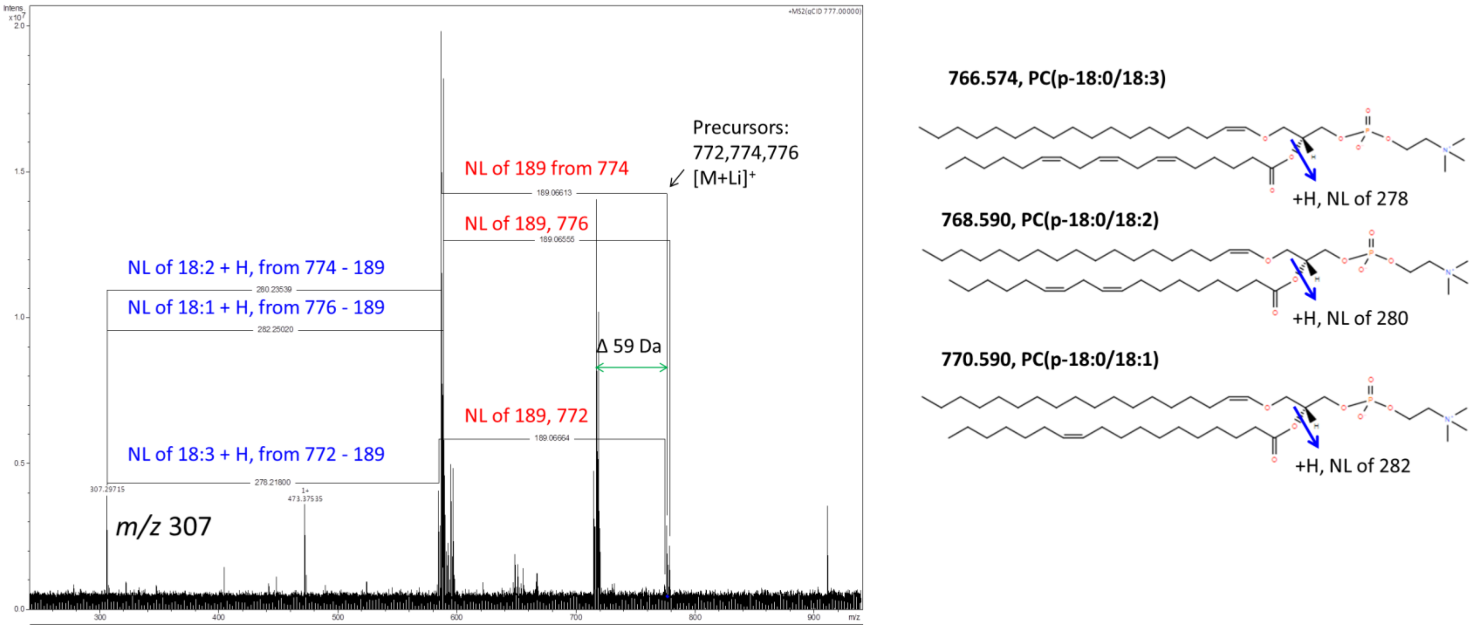

MS/MS of Series 3, isolation window 771.5-777.5. Three precursor ions.

Supplemental Figure 8:

MS/MS of ceramide species co-localized in areas of CRCLM UN (C16 Ceramide) and ILN(Cer-1-P):

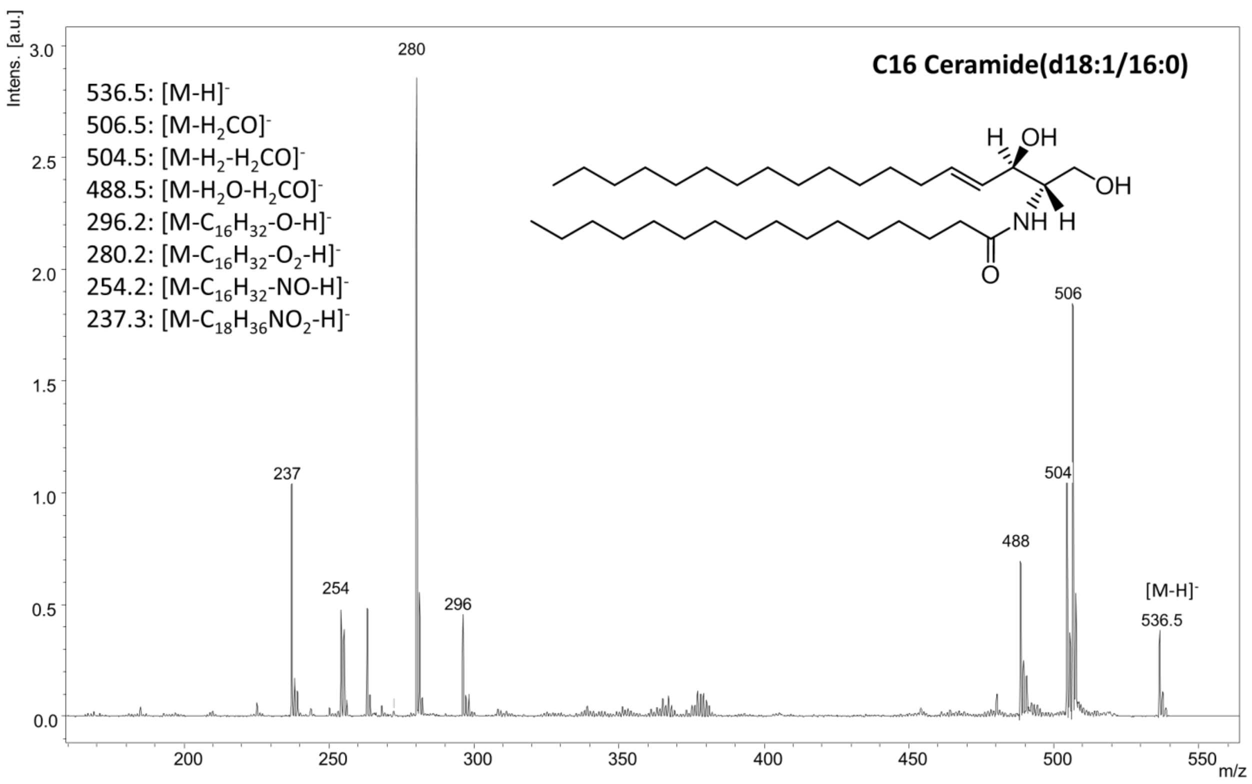

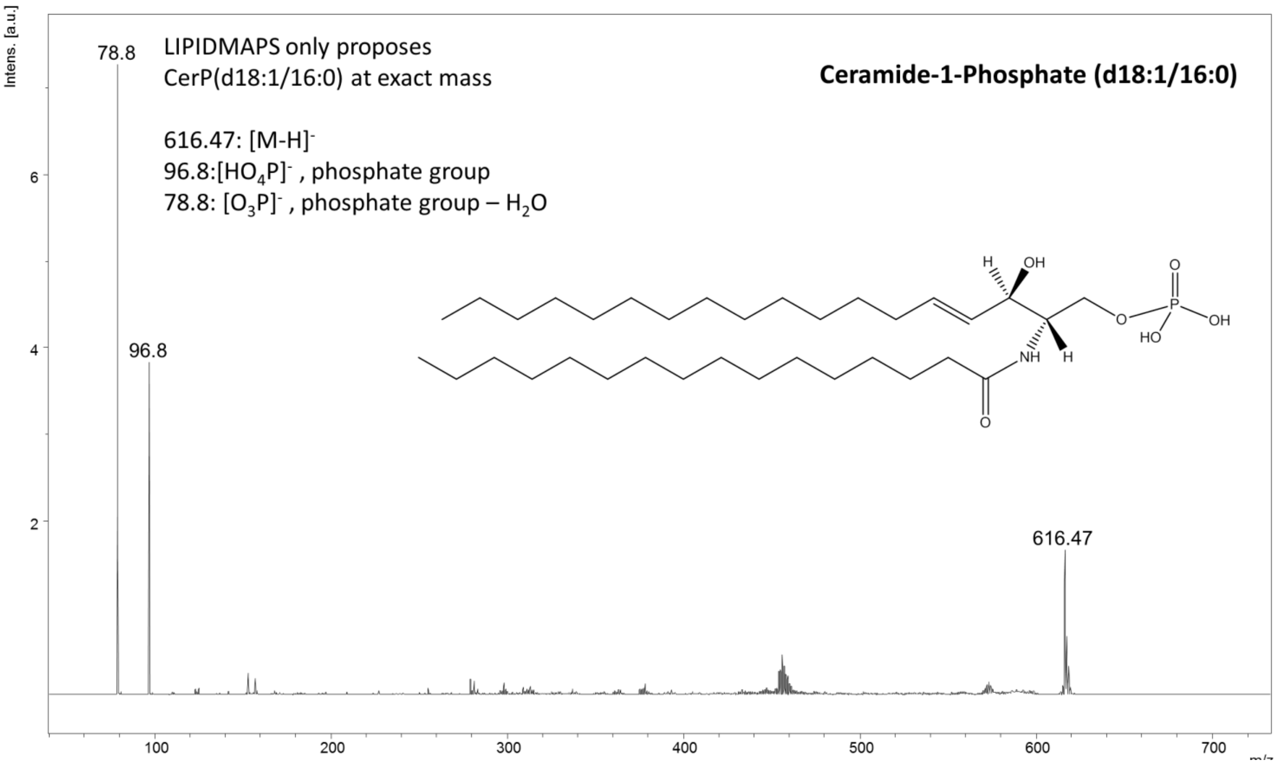

This MS/MS identifies primarily the very labile phosphate grou. For this mass, Cer-1-P is the only entry in LIPIDMAPS and the Human Metabolome Database.

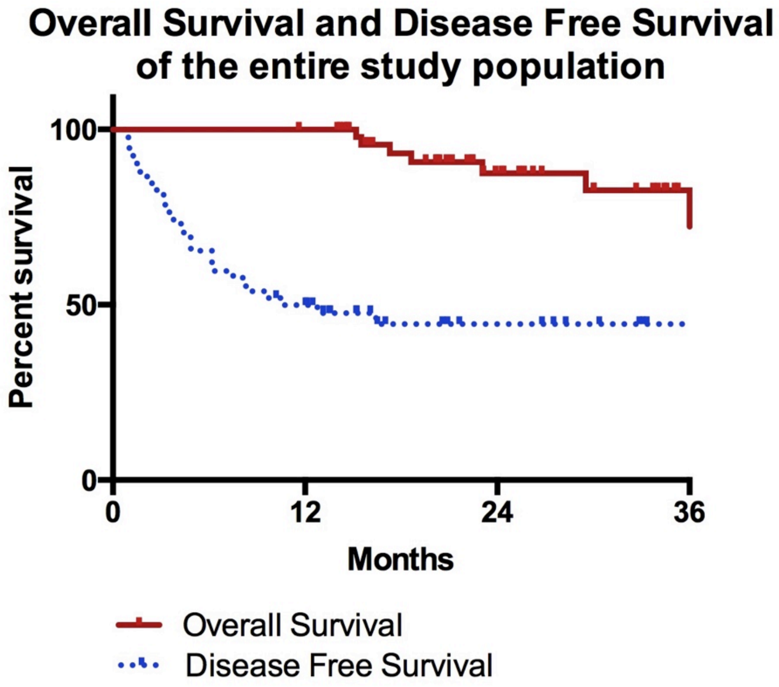

Supplemental Figure 9. Overall Survival (OS) and Disease Free Survival (DFS) of the entire population. Estimated 1-year and 3-year OS were at 100%, and 82.6% respectively (26.5 months mean follow up duration). Estimated 1-year, and 3-year DFS for liver metastasis were at 49.9%, and 44.4% respectively (26.5 months mean follow up duration).
